# The Cinnamyl Alcohol Dehydrogenase Gene Family in Melon (*Cucumis melo* L.): Bioinformatic Analysis and Expression Patterns

**DOI:** 10.1371/journal.pone.0101730

**Published:** 2014-07-14

**Authors:** Yazhong Jin, Chong Zhang, Wei Liu, Hongyan Qi, Hao Chen, Songxiao Cao

**Affiliations:** 1 Key Laboratory of Protected Horticulture of Education Ministry and Liaoning Province, Department of Horticulture, Shenyang Agricultural University, Shenyang, Liaoning, PR China; 2 College of Agriculture, Heilongjiang Bayi Agricultural University, Daqing, Heilong jiang, PR China; Northwestern University Feinberg School of Medicine, United States of America

## Abstract

Cinnamyl alcohol dehydrogenase (CAD) is a key enzyme in lignin biosynthesis. However, little was known about CADs in melon. Five CAD-like genes were identified in the genome of melons, namely *CmCAD1* to *CmCAD5*. The signal peptides analysis and CAD proteins prediction showed no typical signal peptides were found in all *CmCADs* and *CmCAD* proteins may locate in the cytoplasm. Multiple alignments implied that some motifs may be responsible for the high specificity of these CAD proteins, and may be one of the key residues in the catalytic mechanism. The phylogenetic tree revealed seven groups of CAD and melon CAD genes fell into four main groups. *CmCAD1* and *CmCAD2* belonged to *the bona fide* CAD group, in which these CAD genes, as representative from angiosperms, were involved in lignin synthesis. Other *CmCADs* were distributed in group II, V and VII, respectively. Semi-quantitative PCR and real time qPCR revealed differential expression of *CmCADs*, and *CmCAD5* was expressed in different vegetative tissues except mature leaves, with the highest expression in flower, while *CmCAD2* and *CmCAD5* were strongly expressed in flesh during development. Promoter analysis revealed several motifs of CAD genes involved in the gene expression modulated by various hormones. Treatment of abscisic acid (ABA) elevated the expression of *CmCADs* in flesh, whereas the transcript levels of *CmCAD1* and *CmCAD5* were induced by auxin (IAA); Ethylene induced the expression of *CmCADs*, while 1-MCP repressed the effect, apart from *CmCAD4*. Taken together, these data suggested that *CmCAD4* may be a pseudogene and that all other *CmCADs* may be involved in the lignin biosynthesis induced by both abiotic and biotic stresses and in tissue-specific developmental lignification through a CAD genes family network, and *CmCAD2* may be the main CAD enzymes for lignification of melon flesh and *CmCAD5* may also function in flower development.

## Introduction

The lignification of tissues is thought to play a critical role in specialized conducting and supporting tissues of plants, facilitating water transport, providing mechanical strength, and defense against biotic and abiotic stresses [1]–[6]. Lignification is a complex process, which involves many intermediates and enzymes [7]. Cinnamyl alcohol dehydrogenase (CAD) catalyses the final step of the lignin biosynthesis, the conversion of cinnamyl aldehydes to alcohols, using NADPH as a cofactor [8]. Early reports on the identification of CAD enzymes have showed the CAD in gymnosperm is encoded by a single gene [9], [10]. However, CAD is encoded by a multigene family in angiosperm species. Complete sets of CAD genes and CAD-like genes have been identified in the genomes of model species and non-model plants [11]–[25].

Previous studies highlighted that CAD genes in angiosperm species were distributed in different classes according to phylogenetic tree [3], [23]–[27], which were part of a family of related proteins. Recent phylogenetic analysis suggested that the emergence of real lignin in the vascular plant lineage was associated with the origin of *the bona fide* CAD genes [28]. The primary genes involved in lignin biosynthesis,such as *AtCAD4* and *AtCAD5*(*Arabidopsis*) [29], *EgCAD2* (eucalyptus) [30] and *OsCAD2* (rice) [23] have been characterized, which have a dominant role in normal tissue lignification. Furthermore, Other CADs could serve in other functions in different plant tissues. These function could range from a role in redundant (non-xylem) tissue lignification (example as a response to stress) [6], [20]–[22], [31], [32] and in biochemical processes unrelated to lignification [33]. Interestingly, there are differences in function between members of CAD genes family in angiosperm species; However, comprehensive analyses of lignified tissues of various *Arabidopsis* knockout mutants (for *AtCAD5, 6*, and *9*) at different stages of growth/development indicated the presence of functionally redundant CAD metabolic networks [14], [15], [23], [29,]. The biochemical roles of CAD genes still need to be elucidated, particularly being differences in the lignin composition of tissues/plant species result from the different activities of several CAD isoenzymes, each with a different specificity. However, even though CADs have been investigated from a number of plant species, there are currently no reports on CAD in melons.

In this study we identified five CAD-like genes from the melons genome, and compared CAD sequences from a wide variety of plants, making full use of the available plant genome sequences (*Arabidopsis*, Oryza, Sorghum, Wheat, Populus, Medicago, et al.) as well as expressed sequence databases for species of basal angiosperms and gymnosperms. Alignment and phylogenetic analysis of the CAD gene family with related CAD proteins from other species indicated that five *CmCADs* were classified into four groups separately. We analyzed the structure and the promoter of melon CAD genes, and also investigated this CAD genes transcript in response to various fruit development stages and monitored tissue-specific expression. The effect of various plant hormones on *CmCADs* was also examined. We reported here the results of these analyses, suggesting that the *CmCADs* may be involved in melon fruit lignification induced by various hormones and during development and ripening and in tissue-specific developmental lignification.

## Materials and Methods

### Materials and treatments

All the experiments were carried out with ‘CaiHong7’, oriental sweet melons (*Cucumis melo* var. *makuwa* Makino). They were grown in pots (volume of 25 L, soil: peat: compost = 1∶1∶1) in a greenhouse under standard cultural practices for fertilization and pesticide treatments at Shenyang Agricultural University, Shenyang, China, from March to June in 2012. Freshly opened female flowers were sprayed with growth regulator ‘Fengchanji 2’ (a hormone complex, which mainly contains 4-chlorophenoxyacetic acid to increase the rate of fruit set; Shenyang Agricultural University) and tagged on the day of bloom to identify fruit of known age. Plants were trained as single stem, and two or three fruits per vine. Physiological maturity of this sweet melon is about thirty-six days after anthesis. Melons were harvested after 1, 5, 10, 15, 20, 25, 30, 33, 36, 39, 42, 45 and 48 days after anthesis. Mature leaf, developing leaf, pistillate flower, staminate flower, young stems and root tissues were collected from plants grown in a greenhouse for expression analysis.

Mature unripe oriental sweet melons were harvested at 30 days after anthesis (pre-climacteric stage) with the same node of the plant at a mature green stage, before the onset of ripening, and ripening was initiated by exposing the fruits to exogenous ethylene (100 µL/L) for 24 h in a closed 33 L chamber and then allowed to ripen for 12 days at 23°C in air only. Fruits that were allowed to undergo post-harvest ripening in air for 12 days without any exogenous ethylene treatment were treated as control. For 1-methylcyclopropene (1-MCP) (an ethylene perception inhibitor) treatment, mature unripe fruits were exposed to 100 µL/L 1-MCP for 12 h followed immediately by 100 µL/L ethylene treatment for 24 h and then kept at 23°C in air as in earlier cases. Three replicates were prepared for each treatment, and each replicate consisted of 15 fruit unless indicated otherwise. Fresh tissue was sampled every 48 h, frozen in liquid nitrogen and stored at −80°C until further use. For abscisic acid (ABA) and auxin (IAA) treatment, the fruit discs were dipped in a solution containing 100 µM ABA or IAA in 0.2% teepol (detergent) and vacuum infiltrated for 2 h. Infiltrated with 0.2% teepol were used as control fruits. All plant materials were frozen in liquid nitrogen and stored at −80°C. Three replications were carried out for abscisic acid (ABA) and auxin (IAA) treatment or control groups.

### Identification of melon CAD genes

The keyword ‘cinnamyl alcohol dehydrogenase’ was used to search for the melon cinnamyl alcohol dehydrogenase sequences from the melon (*Cucumis melon* L.) genome (http://melonomics.net) [34]. In additional, to further confirm the accuracy of these genes, the predicted CAD-like gene sequences were compared to CAD proteins in other species by a BLASTp retrieve. Only those sequences with high score (>200) were selected.

### Sequence analysis

The CAD protein prediction (amino acid number, calculated molecular weight and isoelectric points) was performed through SIB Bioinformatics Resource Portal (http://web.expasy.org/translate/, http://web.expasy.org/computepi/ and http://expasy.org/). The presence of functional domains were checked via NCBI's Conserved Domain Database (CDD) (http://www.ncbi.nlm.nih.gov/Structure/cdd/wrpsb.cgi), PSIpred and PFP-FunDSeqE prediction server (www.expasy.ch/tools/#proteome; http://www.csbio.sjtu.edu.cn/bioinf/PFP-Fun DSeqE) (which allowed to identify a multi-domain structure in our CAD sequence). Multiple alignments were carried out with other known plant CAD proteins using Clustal W2 program and GENEDOC. Phylogenetic analyses of putative melon CAD proteins were carried out using MEGA5 (http://megasoftware.net) program based on the neighbor-joining method (minimum evolution criterion, bootstrap values performed on 1000 replicates). Promoter analysis was carried out by PLANT CARE program. The sequences were analysed for signal peptides using the SignalP4.1 server (http://www.cbs.dtu.dk/services/SignalP/) [24] and TargetP 1.1 Server. *CmCAD* subcellular localization prediction was performed by WoLF PSORT (http://wolfpsort.org/), CELLO v.2.5 (http://cello.life.nctu.edu.tw/), PSLpre (http://www.imtech.res.in/raghava/pslpred/index.html) and Loctree3 (https://rostlab.org/services/loctree3/). The disulfide bond was predicted in Scratch Protein Predictor server (http://scratch.proteomics.ics.uci.edu/).

### RNA isolation and cDNA synthesis

Total RNA from fruit samples, leaves, stem, root, and flower material, were extracted using ultrapure RNA Kit following the manufacturer's recommendations (Kangwei Biotech, Beijing, China). RNA was suspended in RNase-free water (30 µL), treated with DNAse I (Promega, Madison, WI, USA) at 37°C for 50 min, re-precipitated and concentrated (40 µL). The RNA was measured by the NanoDrop Spectrophotometer ND-1000 and quality was checked by electrophoresis (28S rRNA/18S rRNA ratios). The cDNA was synthesized from total RNA (0.5–1 µg) by using M-MLV RTase cDNA Synthesis Kit following the manufacturer's instructions (Cat#D6130, TaKaRa, Tokyo, Japan).

### Semi-quantitative PCR and real time qPCR

Transcript analysis was carried out by semi-quantitative PCR. For this, semi-quantitative PCR was carried out using cDNA prepared from 500 ng DNA free RNA from different fruit and vegetative tissues. PCR was performed using the gene-specific primers for each gene and *18S*rRNA DNA fragment (148 bp) of melon as an internal control. Specific primers for each CAD gene were designed by Primer3 (http://frodo.wi.mit.edu/) and were listed in Table 1. All primers were designed to avoid detection of conserved regions. The PCR products were checked by agarose gel electrophoresis, and were also sequenced by the company (Sangon Biotech Co.Ltd., Shanghai, China) to confirm primer specificity. Twenty cycles were carried out for *CmCADs*.

**Table 1:** Semi-quantitative PCR and real time PCR primers.

Gene	Primer	Sequence (5-3)
CmCAD1	CmCAD1-F	GAGACGCAAGAAGTATTG
	CmCAd1-R	ACTCAGGCATCTTACTAC
CmCAD2	CmCAD2-F	CTTACACTTACGAACTCAG
	CmCAD2-R	CAACTTCCATCACTTCAC
CmCAD3	CmCAD3-F	CCACAACACATCAACCAT
	CmCAD3-R	CATCCGCTAATCTTGCTTA
CmCAD4	CmCAD4-F	CATTGTTGTTCACGAGAG
	CmCAD4-R	CCTATCACTCCAAGAGATT
CmCAD5	CmCAD5-F	GCTGTTAAGATTGCTAAGG
	CmCAD5-R	GTAATCCATTGTCTCTGTTG
*18*s*rRNA*	18s*rRNA*-F	AAACGGCTACCACATCCA
	18s*rRNA*-R	CACCAGACTTGCCCTCCA

Real time qRT-PCR was performed in a 20 µL reaction volume using SuperReal PreMix Plus (SYBR Green) (Cat.FP205, Tiangen Biotech, Beijing, China) on an ABI PRISM 7500 sequence-detection system according to manufacture's instructions. The gene-specific primers of real time qRT-PCR were the same as that of semi-quantitative PCR. Real-time qRT-PCR conditions were as follows: 50°C for 2 min, followed by 95°C for 10 min, then 45 cycles of 95°C for 15 s and 60°C for 1 min. All real-time qRT-PCR experiments were run in triplicate with different cDNAs synthesized from three biological replicates. Samples were run in triplicate on each 96-well plate. For each sample, a Ct (threshold sample) value was calculated from the amplification curves by selecting the optimal △Rn (emission of reporter dye over starting background fluorescence) in the exponential portion of the amplification plot. Relative fold differences were calculated based on the comparative Ct method using *18S*rRNA DNA fragment (148 bp) of melon as an internal standard. To determine relative fold differences for each sample in each experiment, the Ct values for all *CmCADs* were normalized to the Ct value for *18S*rRNA and was calculated using the formula 2^−△△Ct^. The means of *CmCADs* expression levels were calculated from three biological repeats, obtained from three independent experiments.

### Statistical analysis

Data are expressed as mean values ± standard deviation of three independent experiments (n = 3). The data were analyzed by the analysis of variance (ANOVA) using the SPSS 13.0 statistics program, and significant differences were compared by a one-way ANOVA following Duncan's multiple range tests for each experiment at a P<0.05 level. The charts were generated by using Origin (version 8.0).

## Results

### Identification of melon CAD genes

All the predicted CAD genes in the melon genome were collected and compared with CAD genes in other species. In this case, the presence of functional domains was checked via NCBI's Conserved Domain Database (CDD) (http://www.ncbi.nlm.nih.gov/Structure/cdd/wrpsb.cgi), and only those sequences having the same functional domains of CADs with other proved/putative species were selected as our target genes. Based on this information, 5 CAD genes, encoding full-or nearly full-length functional proteins, were identified in the melon genome. By ExPASy tools, we found that the longest protein of CADs consisted of 360 amino acid residues, and the shortest consisted of 355 amino acid residues. The ORF length ranged from 1068 to 1083 nucleotides. The predicted molecular weight and isoelectric points of all CADs proteins ranged from 38.42 kDa/5.68 to 39.62 kDa/6.63, respectively (Table 2). For unreported CAD genes in melon, we gave them a name by adding a number to their family name in the order in which they were searched.

**Table 2:** The information of CAD genes in melon.

Gene	Gene accession No.	amino acid (aa)	ORF length	Melon predicted protein No.	Molecular weight (KD)	location	Isoelectric point
CmCAD1	MELO3C019548	357	1074	MELO3C019548 P1	39.20	CM3.5_scaffold00038	5.96
CmCAD2	MELO3C018492	356	1071	MELO3C018492 P1	39.40	CM3.5_scaffold00034	5.68
CmCAD3	MELO3C003735	360	1083	MELO3C003735 P1	39.62	CM3.5_scaffold01596	6.63
CmCAD4	MELO3C005809	355	1068	MELO3C005809 P1	38.42	CM3.5_scaffold00005	5.99
CmCAD5	MELO3C023272	358	1077	MELO3C023272 P1	38.87	CM3.5_scaffold00059	6.14

### Intron-exon structure of melon CAD genes

**Figure 1:**
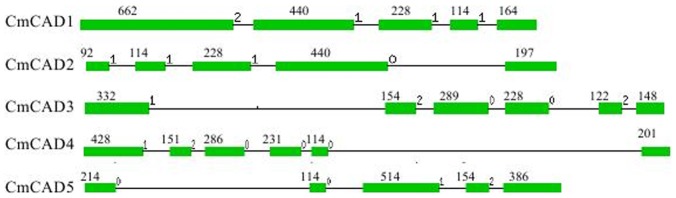
Intron-exon structures of CAD genes from melon. Exons and introns are indicated by open boxes and lines respectively. Numbers above boxes indicate the exon sizes. The intron sizes are not to scale. The names of CAD genes and intron-exon structure are indicated at the left and right sides respectively.

Structural analysis of the identified melon CAD genes revealed different intron-exon patterns both in relation to position and number of introns, which ranged from four to five per gene (Figure 1). Furthermore, substantial differences in the size between the exons were observed. Based on Figure 1, one individual intron may be missing in *CmCAD1*, *CmCAD2* and *CmCAD5*, because introns located exactly at the same position have been present in the common ancestor [26]. Hence, the position and type of the introns should be strictly conserved between the different genes when present. Of the five identified introns, only *CmCAD3* and *4* contain all five. Additionally, A 114-bp sequence was found in four sequences except *CmCAD3* (Fig. 1). According to the model proposed by Youn et al. [35], this 114-bp sequence in *CmCAD2* and *CmCAD5* should encode the putative binding site-I for the monolignol substrate. The 114-bp sequence was identical to the second exon flanked by introns in *CmCAD2* and *CmCAD5*. Relative to these, this sequence was identical to the fourth exon in *CmCAD1* and *CmCAD4*, and it was unclear whether this sequence encoded the putative binding site.

### Phylogenetic analysis and characterization of the *CmCAD* genes family

**Figure 2:**
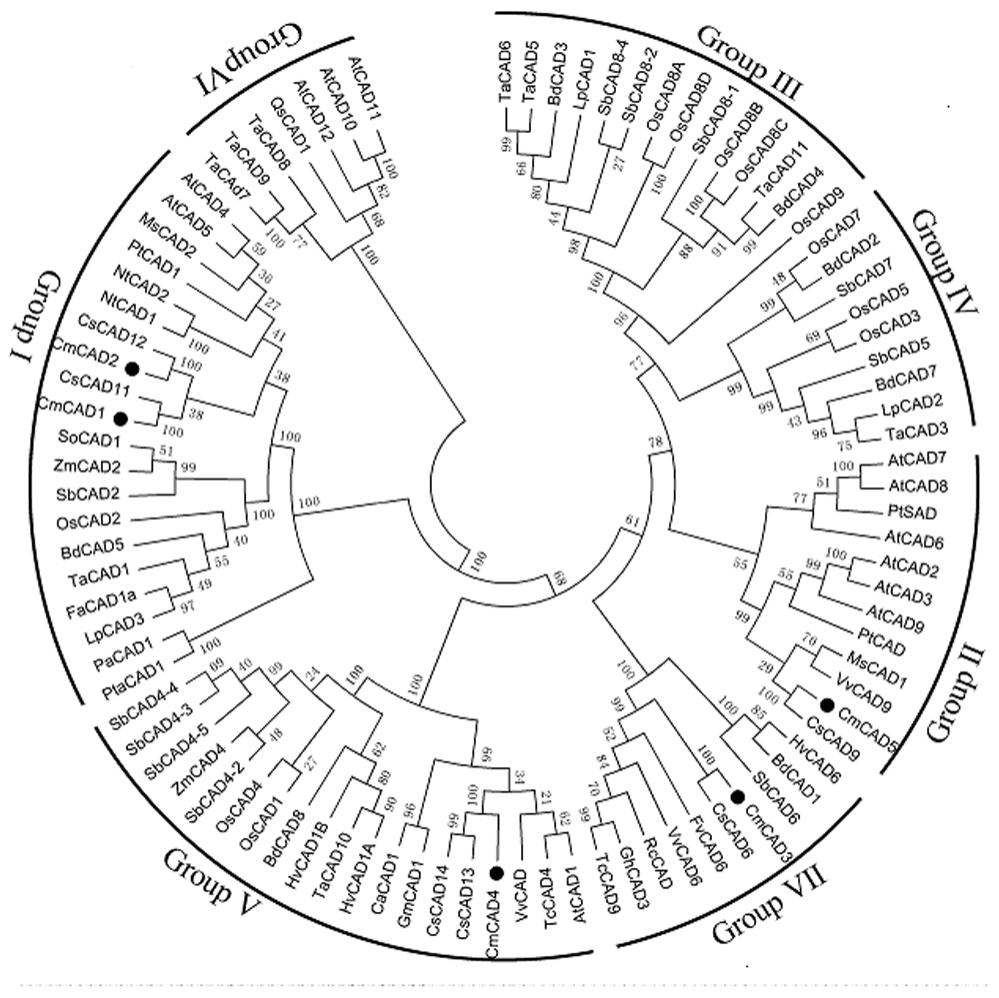
Phylogenetic relationship among CmCADs and CADs from other plant species. The amino acid sequences were aligned by the Clustal W2 program, and the neighborjoining tree was drawn with TreeView. The corresponding GenBank and the melon genome (https://melonomics.net/) were noted in the phylogenetic tree and the accession number in the melon genome were CmCAD1 (MELO3C019548P1), CmCAD2 (MELO3C018492P1), CmACD3 (MELO3C003735P2), CmCAD4 (MELO3C005809P1) and CmCAD5 (MELO3C023272P1). The number for each interior branch was the percentage of bootstraps value (1000 replicates). Black circle denoted five CmCADs. CADs belong to the following plant species: *Arabidopsis thaliana*: AtCAD1 (AY288079), AtCAD2 (AY302077), AtCAD3 (AY302078), AtCAD4 (AY302081), AtCAD5 (AY302082), AtCAD6 (AY302075), AtCAD7 (AY302079), AtCAD8 (AY302080), and AtCAD9 (AY302076); *Oryza sativa*: OsCAD1 (AAN09864), OsCAD2 (DQ234272), OsCAD3 (AAP53892), OsCAD4 (BK003970), OsCAD5 (BK003971), OsCAD6 (CAD39907), OsCAD7 (CAE05206), OsCAD8A to D (BK003972), and OsCAD9 (AAN05338); *Sorghum bicolor*: SbCAD2 (Sb04g005950), SbCAD4-2 (Sb10g006300), SbCAD4-3 (Sb10g006290), SbCAD4-4 (Sb10g006280), SbCAD4-5 (Sb10g006270), SbCAD5 (Sb07g006090), SbCAD6 (Sb06g001430), SbCAD7 (Sb06g028240), SbCAD8-1 (Sb02g024220), SbCAD8-2 (Sb02g024210), and SbCAD8-4 (Sb02g024190); *Triticum aestivum*: TaCAD1 (GU563724), TaCAD2 (TC143210), TaCAD3 (TC143265), TaCAD4 (TC144004), TaCAD5 (TC149391), TaCAD6 (TC149393), TaCAD7 (TC170425), TaCAD8 (TC170426), TaCAD9 (TC170429), TaCAD10 (TC172690), and TaCAD11 (TC179401); *Aralia cordata*: AcCAD1 (D13991); *Eucalyptus globulus*: EgCAD1 (AF038561); *Festuca arundinacea*: FaCAD1a (AF188292); *Lolium perenne*: LpCAD1 (AF472591), LpCAD2 (AF472592), and LpCAD3 (AF010290); *Medicago sativa*: MsCAD1 (AF083333) and MsCAD2 (AF083332); *Nicotiana tabacum*: NtCAD1 (X62343) and NtCAD2 (X62344); *Picea abies*: PaCAD1 (X72675); *Populus tremuloides*: PtCAD1 (AF217957) and PtSAD (AF273256); *Pinus taeda*: PtaCAD1 (Z37992); *Saccharum officinarum*: SoCAD1 (AJ231135); *Zea mays*: ZmCAD1 (AJ005702) and ZmCAD2 (Y13733); *Vitis vinifera*: VvCAD (CBI34634.3), VvCAD6 (XP002269356.1), VvCAD9 (XP002279832.1). *Cucumis sativus*: CsCAD6 (XP004136373.1), CsCAD9 (XP004150677.1), CsCAD11 (XP004140716.1), CsCAD12 (XP004137094.1), CsCAD13 (XP004145884.1), CsCAD14 (XP004162965.1), *Hordeum vulgare*: HvCAD1A (BAJ84795.1), HvCAD1B (BAJ98188.1), HvCAD6 (BAK01962.1); *Glycine max*:GmCAD1 (XP003543132.1); *Cicer arietinum*: CaCAD1 (XP004485621.1); *Gossypium hirsutum*: GhCAD3 (ACQ59091.1); *Ricinus communis*: RcCAD (XP_002510582.1); *Theobroma cacao*: TcCAD9 (EOY15101.1); *Fragaria vesca*: FvCAD6 (XP004291336.1).

To understand the possible functional divergence of the individual members of melon CAD genes family, phylogenetic analysis was performed using deduced amino acid sequences of melon CAD genes and CAD genes from other species. The phylogenetic tree revealed seven groups of CAD, and *CmCADs* were classified into four groups (Figure 2). *CmCAD1* and *CmCAD2* were positioned in the *bona fide* groupI, all of which have been characterized as *bona fide* CAD genes (groupI) involved in lignin biosynthesis [3]. *CmCAD5* belonged to groupII; *CmCAD4* clustered into groupV; *CmCAD3* belonged to groupVII.

**Figure 3:**
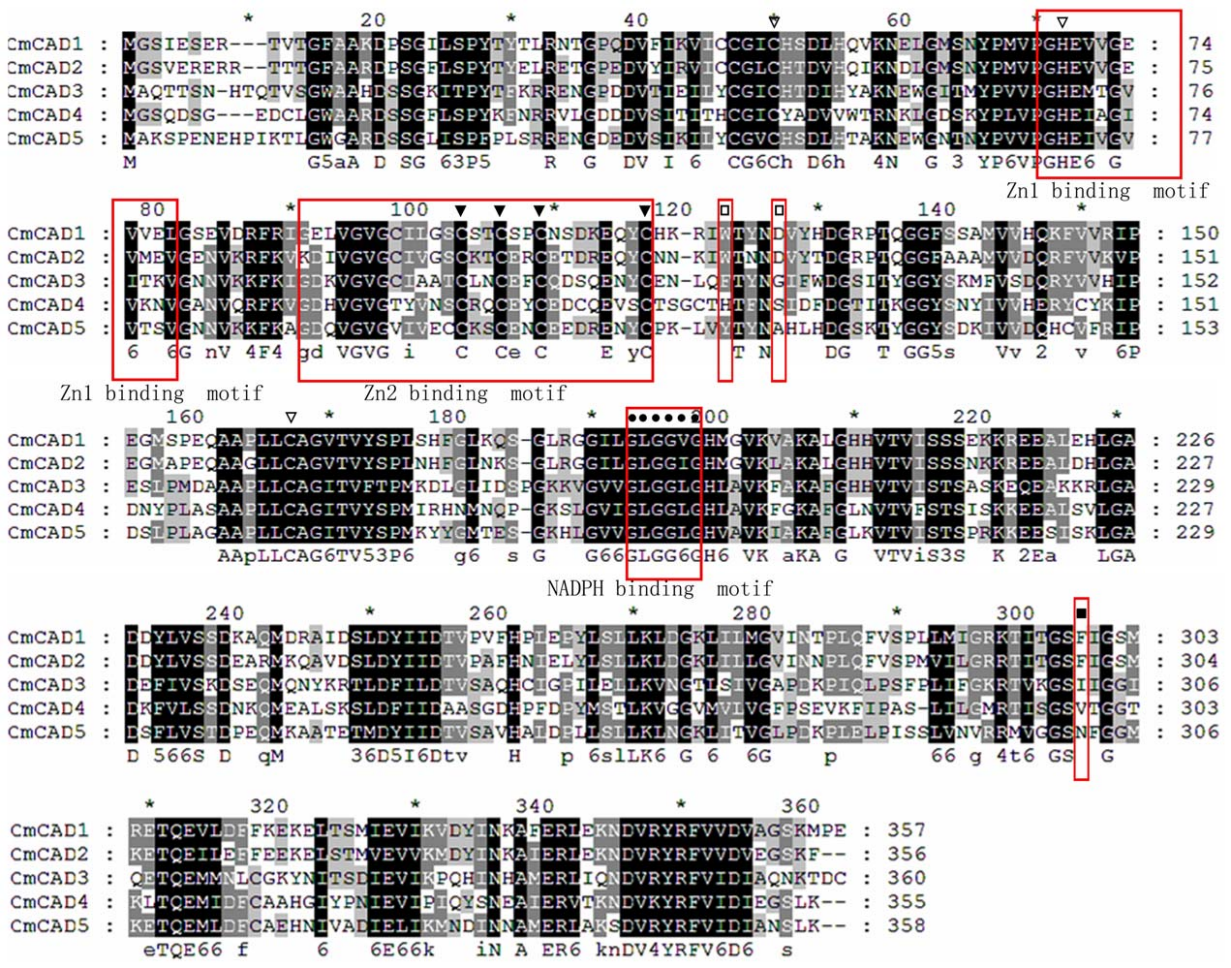
Alignment of amino acid sequences of CmCADs. Conserved important regions identified previously are marked as follows: white arrows denotes catalytic zinc ion coordinating residue, black arrows denotes structural Zn ion coordinating residue, the black circle denotes key residues for substrate specificity. The black square denotes key Phe299/Gly(300) residues for substrate specificity. The white square denotes key Trp199 and Asp123 residues for substrate binding. Locations of the Zn1, Zn2, and NADPH binding domains are shown in boxes. The alignment was performed with the ClustalW2 software program.

Multiple alignments revealed that the deduced amino acid sequence of *CmCAD1* showed the highest homology with a CAD (GenBank no. ADO16245.1) in *Ocimum tenuiflorum* (99.17% identity); Other *CmCADs* showed the highest homology with corresponding CADs from *Cucumis sativus* (GenBank) (Figure S4–S7). The protein sequences of melon CAD genes were aligned against each other (Figure 3). However, only 77.07% identity was found between *CmCAD1* and *CmCAD2*, and there were lower identity between *CmCADs*. All of the melon CADs had the highly conserved Zn1 catalytic center (C47, H69, and C163), the Zn-binding signature GHE(X)2G(X)5G(X)2V, the Zn2 structural motif (C100, C103, C106, and C114), and the NADPH-binding domain [GLGGV(L)G] motif (so-called Rossmann fold) (Figure 3), suggesting that these proteins appear to be zinc-dependent alcohol dehydrogenases and members of the plant CAD protein family and the medium-chain dehydrogenase/reductase (MDR) superfamily [36], [37].

Out of all amino-acid residues in five *CmCADs*, 109 were invariant (30% identity). Phe299/Gly300 is a key determinant residue of substrate specificity [21], [38], and Trp119 and Asp123 are key residues in an optimal position for substrate binding [39], which were found in the sequence of *CmCAD1* and *CmCAD2*. Additionally, previous researches also showed a few critical residues of identifying lignifying CADs, including His/Asp57, Leu58, Glu70, Cys/Ile/Val95, Ser120 and Ser212 [35], [40]. *CmCAD1* and *CmCAD2* had most of these critical residues, compared with other *CmCADs* (Figure 2). In *Brachypodium*, the amino-acid residues 58HL59 in the *BdCAD5* sequence, believed to be part of the catalytic mechanism as a proton donor [24], were not found in *CmCADs* which contain either EW or DL/EL dipeptide, except for *CmCAD4* which contains KL, indicating a change from E to K as a result of a G→A base transversion. This change in one of the proton-donating residues according to the model of Youn et al. [35] may lead to an inactive *CmCAD4*. Compared with the HL or DL motif present in monocot and eudicot CADs, increasing activity on a range of monolignals, an equivalent EW sequence motif at the H57/L58 positions of *CmCAd3* and *CmCAD5* may alter apparent substrate preferences [39].

No typical signal peptides were found in all *CmCADs* after analyzing their N-terminals using the SignalP software. *SbCAD6* has an evolutionarily conserved SKL sequence at the C-terminus, which may serve as a signal peptide sequence to locate these enzymes in plant cell peroxisomes [17]. But no homology was found between the SKL sequence of *CmCADs* and those of other species through amino acids sequence comparison, suggesting that *CmCADs* may not be located in peroxisomes [17]. *CmCADs* subcellular localization prediction showed that these CAD genes may exist in the cytoplasm (Table S2). Moreover, the transmembrane topology predictions of five *CmCADs* using TMHMM 2.0 software and ABTMpro showed that there was no internal transmembrane segment in *CmCADs*. We also found there were 4 and 3 disulfide bonds in *CmCAD1*, *CmCAD2*, *CmCAD3* and *CmCAD4* proteins by Scratch protein Predictor, respectively, but there were 3 disulfide bonds in *CmCAD5* protein.

### Promoter sequence analysis

Analysis of promoter sequences of the melon CAD genes allowed us to identify several motifs that were known to be involved in the regulation of gene expression in various developmental and physiological processes. A promoter motif search showed that *CmCAD* promoter contained putative regulatory elements corresponding to known cis-elements of eukaryotic genes [19]. In melon CAD genes promoter, there were mainly two kinds of motifs, namely, cis-acting element involved in defense and stress responsiveness (such as hypoxia stress, heat stress) and cis-acting regulatory element involved in the response to various hormones, including some of these motifs involved in responses to biotic and abiotic stresses, such as auxin (IAA), ethylene, abscisic acid (ABA), salicylic acid (SA), and methyl jasmonate (MeJA) (Table S1) [19]. *CmCAD3* and *CmCAD4*, which possess some motifs involved in stress responsiveness and in the response to various hormones, showed some differences in their sets of motifs. For instance, *CmCAD3* also has motifs involved in response to wound and fungal elicitor responsive element, while *CmCAD4* also possess MYB binding site involved in drought-inducibility. Other genes (*CmCAD1, CmCAD2*) possess elicitor responsive element and enhancer. These results indicated that transcriptional regulation of these *CmCAD* genes may be involved in fruit development and in the response to various stresses.

### *CmCADs* expression in vegetative tissues

**Figure 4:**
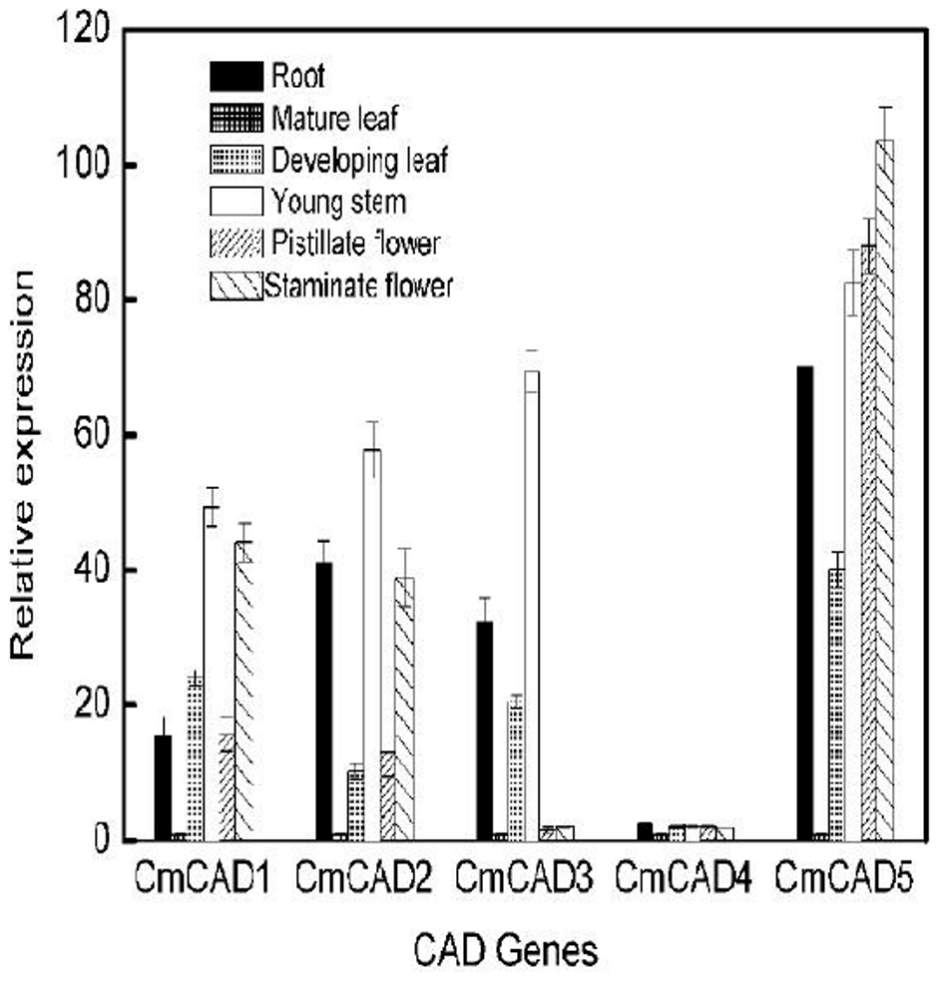
Transcript levels of these five CmCAD in different melon organs. The gene expressions of *CmCAD* in different organs in melon plants were determined by qRT-PCR in root, developing leaves, mature leaves, young stems, pistillate flower petals and staminate flower petals in melon plants. *18* *s* were used as internal control. The expression level of the genes in mature leaves was set as “1.0”. Data represent the means±SD (n = 3) of three biological samples. The experiments were repeated 3 times with similar results.

In order to investigate the transcript levels of these five *CmCADs* in different organs in melon plants, we collected samples of root, developing leaves, mature leaves, young stems, pistillate flower petals and staminate flower petals. Expression analysis using semi-quantitative PCR and real time qPCR showed that four of these CAD genes were expressed in these organs, but greatly varied in different tissues (Figure 4; Figure S1). Of the five CAD genes identified in melons, *CmCAD5* expression was greatest in vegetative organs except mature leaves, in which *CmCAD2*, *CmCAD3* and *CmCAD5* were weakly expressed. *CmCAD4* was either not expressed or expressed at very low levels in these tissues. Lignin biosynthesis genes are expected to be highly expressed in stems, where secondary cell walls are prevalent and lignification occurs, while remaining at relatively low levels in roots and especially leaves [41]. As might be expected, apart from *CmCAD4*, the other *CmCAD* were expressed at a slightly lower level in roots than in young stems, where lignin is also present, but these four *CmCAD* genes were highly expressed in developing leaves. Furthermore, *CmCAD1*, *CmCAD2* and *CmCAD3* showed significant expression differences between pistillate flower petals and staminate flower petals, with higher transcript levels in staminate flower petals.

### *CmCADs* expressions during melon fruit development

**Figure 5:**
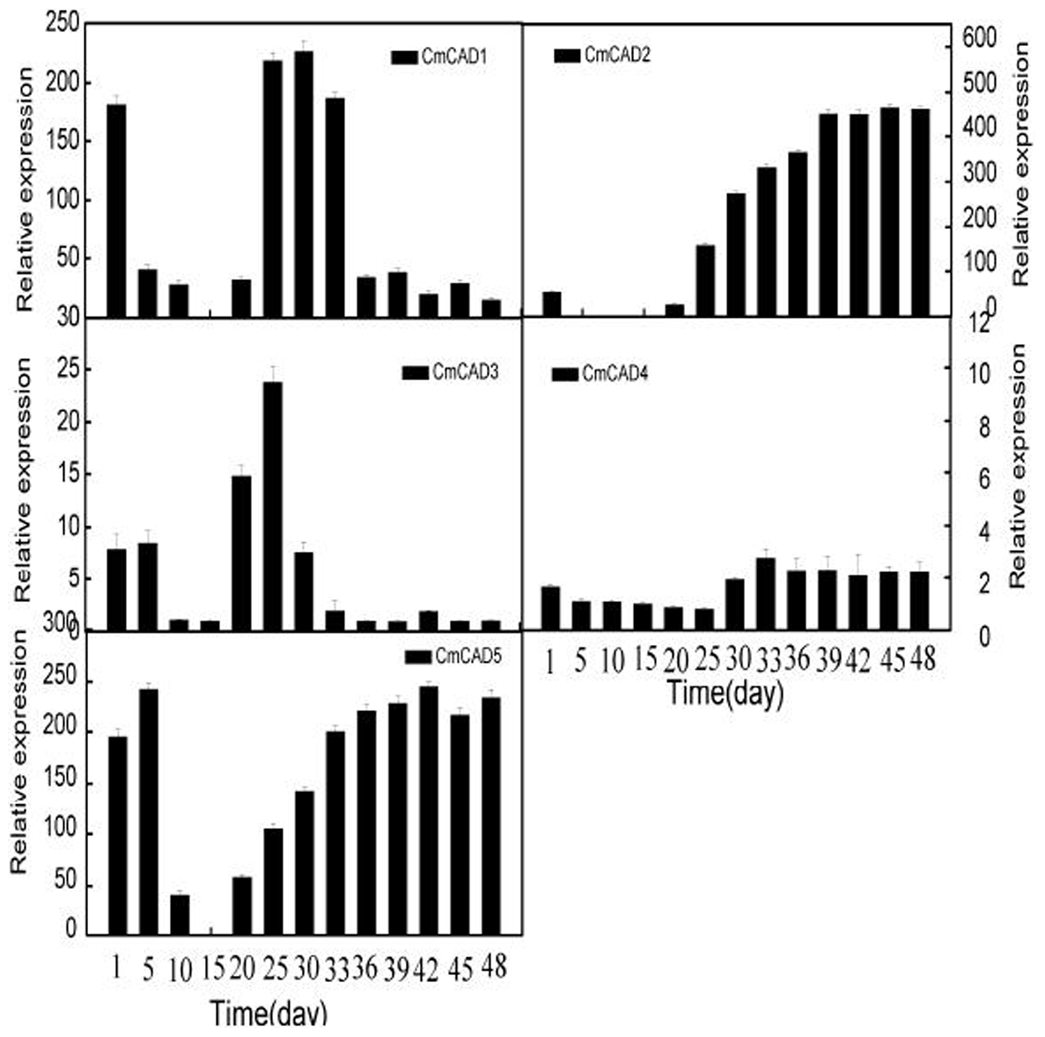
CmCADs relative expression in developing stages of melon fruit after pollination were determined by qRT-PCR. *18* *s* were used as internal control. The expression level of *CmCADs* in melon fruit at 15days after pollination was set as “1.0”. Data represent the means±SD (n = 3) of three biological samples. The experiments were carried out in triplicate.

In the present study, five *CmCAD* expressions were analysed during melon fruit development. Real time qPCR and semi-quantitative PCR analysis indicated that these five *CmCAD* genes studied here were specifically expressed in fruit (Fig.5; Figure S1). The pattern of changes in transcript levels was similar for *CmCAD1*, *CmCAD3* and *CmCAD5* from 1 to 15days after anthesis with transient and sharp decrease at 15 days; while the expression of *CmCAD2* was only expressed at 1 day. The expression of *CmCAD1* and *CmCAD3* showed an increase after 15 days, and gradually reduced after 30 days. In contrast, *CmCAD2* and *CmCAD5* consistently had higher expression in fruit after 30 days, with an increase in transcript abundance, which subsequently remained at a relatively constant level through to harvest. *CmCAD4* was either not expressed or expressed at very low levels during fruit development.

### Effects of hormones on *CmCAD* genes expressions

**Figure 6:**
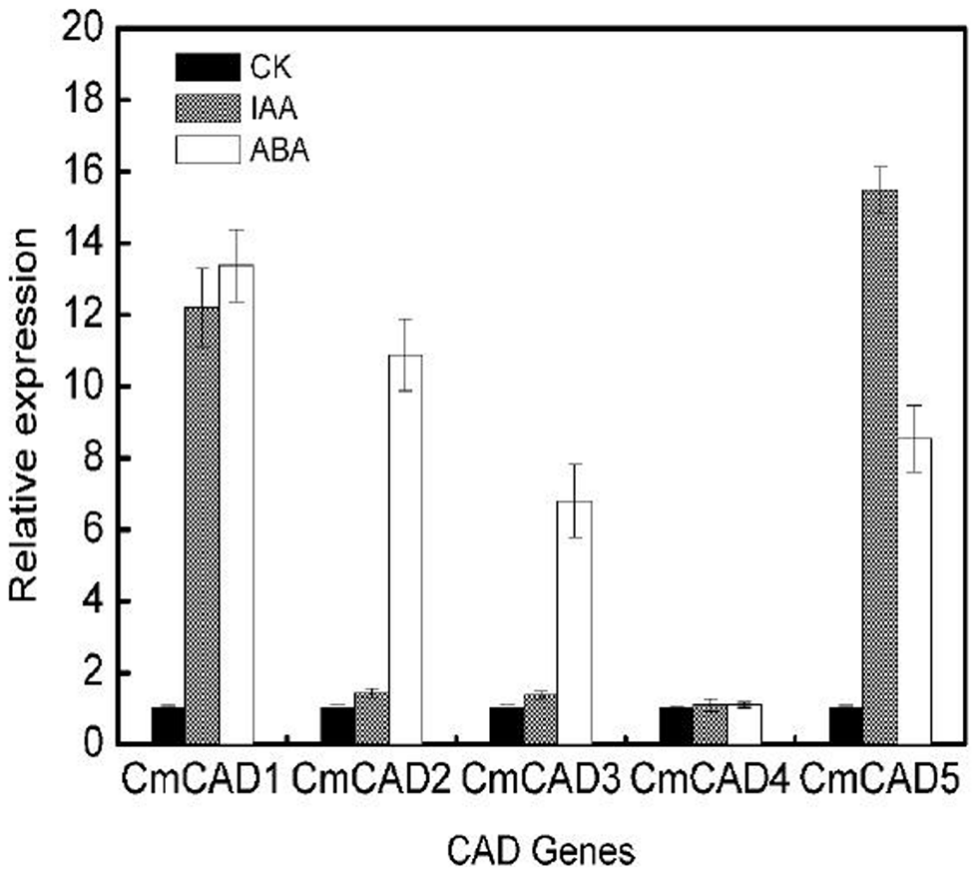
The expression of *CmCAD1*, *2*, *3*, *4* and *5* in melon fruit after different hormonal treatments. IAA and ABA (100 µM) treatments were given for 3 h as described in materials and methods section. Expression analysis was carried out by real time qPCR. For each gene, the relative abundance of mRNA was normalized against the *18S* in the corresponding samples. The expression level of the genes in untreated melon fruit by IAA and ABA was set as “1.0”. Data represent the means±SD (n = 3) of three biological samples. The experiments were repeated 3 times with similar results.

Since ripening is also affected by other hormones like ABA and IAA, we studied the regulation of these genes in fruits by treatment with ABA and IAA. The real time qPCR and semi-quantitative PCR results revealed that *CmCADs* were induced by ABA (though expression was lower in case of ABA), except *CmCAD4* (Figure 6; Figure S2). In contrast, IAA treatment strongly induced *CmCAD1* and *CmCAD5* expression. Other *CmCADs* were seemed to be insensitive to treatment with IAA and continued to be weakly expressed at a basal level.

### Effects of ethylene on *CmCADs* genes Expression

**Figure 7:**
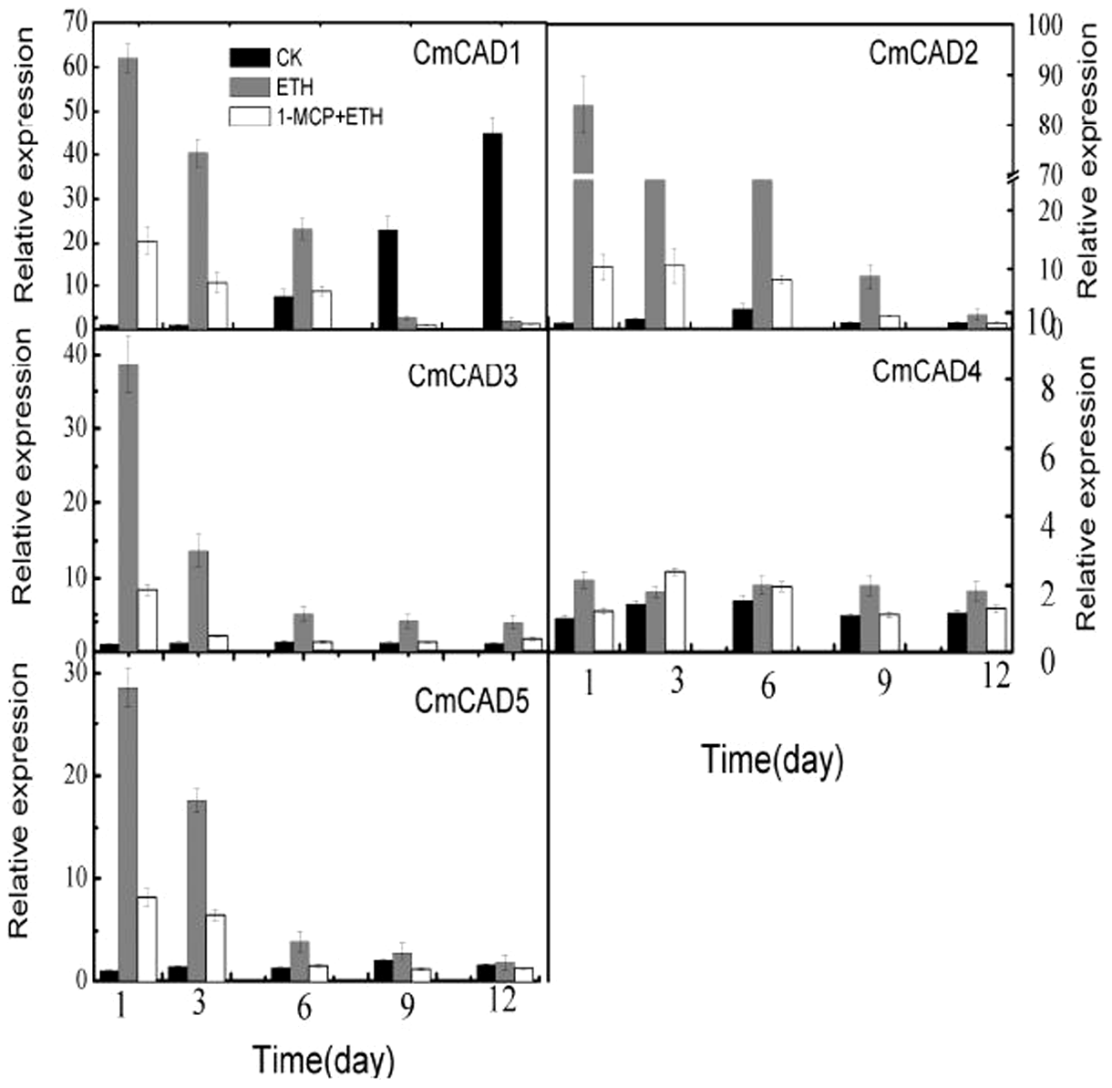
The expression levels of CmCADs after treatment with ethylene and 1-MCP. The transcript levels of *CsCADs* were measured by real time qPCR in melon fruit treated, and *18S* were used as internal control. The expression of the genes in untreated melon fruit after 1 day of storage was set to 1.0. Data represent the means±SD (n = 3) of three biological samples. The experiments were repeated 3 times with similar results.

Since oriental sweet melons is a climacteric fruit which requires ethylene for initiation of ripening, we checked whether these five CAD genes were regulated by ethylene. Transcript accumulation of different CADs in melon was investigated during the course of ethylene induced ripening of harvested melon for 12 days. A basal level of expression of *CmCADs* transcript was detected in case of all the five CADs in ethylene untreated fruit on day 1. The transcript levels of *CmCADs* shot up on day 1 in ethylene treated fruit, apart from *CmCAD4* (Figure 7; Figure S3). However, levels of *CmCAD1*, *CmCAD2*, and *CmCAD5* transcript gradually decreased from day 1 levels in subsequent days, but levels of *CmCAD3* rapidly reduced, and maintained throughout the progression of ripening thereafter. Of the five, *CmCAD4* transcript showed the lowest steady state levels in all of treatments during the course of melon ripening. We checked the expression of *CmCAD4* transcripts by semi-quantitative PCR at high annealing temperature using gene-specific primers to ensure that the transcript patterns were not due to cross hybridization. We found that *CmCAD4* transcript levels were still lower and did not give clear results when 20 cycles of PCR were carried out as in case of other CAD genes. So for all semi-quantitative experiments related to *CmCAD4*, 35 cycles of PCR were carried out. Our results of semi-quantitative PCRs matched with real time qPCR analysis and confirmed that all the five CADs were expressed at different levels during melon ripening. The expressions of *CmCADs* were also studied in 1-MCP + ethylene treated fruits. All the five *CmCADs* showed transcript accumulation in 1-MCP + ethylene treated fruits, but the levels were far lower compared to the levels in ethylene treated fruits except *CmCAD4*, especially on day 1 post treatment (Figure 7; Figure S3). Since the patterns were same in real-time qRT-PCR and semi-quantitative PCR experiments, further studies needed to be carried out.

## Discussion

Cinnamyl alcohol dehydrogenase (CAD) is a major rate-limiting enzyme in lignin biosynthesis, and it catalyzes the conversion of coniferaldehyde, *p*-coumaraldehyde, and sinapaldehyde to guaiacyl, *p*-coumaryl, and syringyl monolignols, respectively. More importantly, the CAD gene family is related to stress responses during plant growth and is also closely associated with vegetative tissue, flower, pollen grains, fruit and seed maturation and aging [21], [42]. The CAD/CAD-like gene family has been investigated in a number of plant species such as wheat [3], tobacco [11], [13], *Arabidopsis* [14], [15], white spruce [16], sorghum [17], [18], populus [19], [20], sweet potato [21], *brachypodium distachyon* [24], tea [25], rice [26], [43], switch grass [44] and flax [45]. The aim of the present study was to identify and analyse genes coding for the CAD enzyme in melon.

### Expression analysis in different tissue

In angiosperm, multiple homologous CAD genes, thought to have distinct roles, may participate in lignin biosynthesis in different tissues or during different growth and development of one type of tissue [24], [25]. In angiosperm and gymnosperm, expression analysis of CAD genes studied showed that they had different expression profiles. In *Brachypodium distachyon*, *BdCAD5* was the only gene to be expressed in all tissues, with the highest expression in root and stem, and showed high sequence similarity to CAD genes biochemically characterized as *bona fide* CADs; Other *BbCADs* also were expressed in lignified and non-lignified tissues, but the relative expression varied across tissues [24]. *CmCADs* also showed different pattern in tissues and fruits during different developmental stages (Figure4 and 5; Figure S1). Except *CmCAD4*, the other *CmCADs* was highly expressed in young tissues (including developing leaves and young stems), with the same as results in *ginkgo biloba* [42], sweet potato [21] and tea [25]. Additionally, *CmCAD5* had higher expression in flower, and we also found that *CmCAD5* had an equivalent EW sequence motif, altering apparent substrate preferences, at the H57/L58 positions [39], but *CmCAD5* lacked critical residues at the Trp119 and Asp123 positions, implying that *CmCAD5* may participate in flower development or volatile synthesis. In *Arabidopsis thaliana*, *AtCAD4*, *AtCAD5, AtCAD7* and *AtCAD8* participated in lignin biosynthesis, but *AtCAD4* was strongly expressed in flowers and roots; In contrast, the *AtCAD5* gene was expressed in lignified roots and strongly expressed in pathogen-infected tissues [15], [19], [29]. *OsCAD2* and *SbCAD2* have been shown to be responsible for lignin biosynthesis, being related to hull and internode phenotypes in rice [46] and sorghum [18], respectively. These findings suggested that there seems to be the question of their functional redundancy in the CADs genes network. Therefore, we speculated that *CmCADs* might be involved in both lignin synthesis and pathogen defense. These results also suggested that plant CAD genes might also participate in other unknown functions or have evolved individual functions [42]. There is much to learn about CADs in dicotyledon.

Apart from lignin biosynthesis, biotic and abiotic stresses, plant CAD genes were also implicated in fruit tissue development and were closely related to tissue ageing. For example, loquat flesh tissue undergone lignification during ripening, which resulted to the increase in firmness, and CAD transcripts particularly accumulated during storage, modified by ethylene [47]. In the present study, the expression pattern of *CmCADs* differed greatly during fruit development, with *CmCAD1, 2* and *3* weakly expressed or not expressed in early stages of development and *CmCAD1* clearly expressed from 25 to 33 days after the anthesis, whereas *CmCAD5* and *CmCAD2* were strongly expressed during fruit development, apart from 15days after the anthesis (Figure 5; Figure S1). Hence, we speculated that both *CmCAD2* and *CmCAD5* could be involved in fruit development. While *CmCAD2* only belonged to group I as *bona fide* CADs, and may be likely main candidate gene for lignin biosynthesis in melon. However, little information is available on the role of CAD in relation to the lignification of melon flesh tissue during fruit development and ripening. These findings implied that melon CAD genes might also be involved in the lignification of flesh tissue, and there were difference in function among family members.

There were significant expression differences between *CmCAD4* and other *CmCADs* in different tissues and during development and ripening. These observed differences could partly be explained by the amino acids differences at position 58–59 (Figure 3). *CmCAD4* had a KL motif, whereas other *CmCADs* had an either EW or DL/EL motif. Furthermore, based on our analysis of *CmCADs*, *CmCAD4* had little key residues, and had a Ser123 instead of Asp123 which was suggested to be involved in determining the activity of all *bona fide* lignifying CADs; One mutation, D123S, resulted in a essentially inactive protein [39]. On the basis of the above results, it appears that *CmCAD4* is a pseudogene. But we used the expressed sequence tag (EST) database in NCBI as a source of mRNA sequence bioinformatics for *CmCAD4*, and we found that *CmCAD4* showed the highest homology with a *Cucumis melo* cDNA clone (GenBank no. JG465149.1) (92% identity), obtained from callus, from EST database of melon (CM-PEa library). Hence, It appears that *CmCAD4* is specifically expressed in callus. Further researches are needed to confirm this speculation.

### Induced expression analysis

We also studied the effect of ABA and IAA on *CmCADs* expression. ABA is extensively involved in the plant's response to abiotic stresses, such as drought, low temperature and osmotic stress, and also regulates a variety of growth and developmental processes, and can regulate the expression of relevant genes to increase plant adaptability [48]. ABA has been shown to induce CADs genes expression in sweet potato [21], ginkgo [42] and tea [25] after exposure to biotic or abiotic stresses. Real time qPCR and semi-quantitative PCR analysis of *CmCADs* in response of ABA suggested that ABA treatment increased the expressions of *CmCAD1, 2, 3* and *5* (Figure 6; Figure S2). Purportedly, ABA-mediated plant responses to drought stress may be related to the regulation of relevant genes by the MYB transcription factor [44]. However, to the best of our information, there were no reports on effect of ABA or IAA on ripening specific CADs. Promoter analysis of the five melon CADs suggested the presence of ABA responsive ABRE motif [49] in the promoter of *CmCAD1*, *2*, 3 and *5*, and the promoter of *CmCAD1* and *CmCAD5* contains response elements (TGA) to IAA (Table S1). Therefore, the ABA-induced these *CmCADs* expression observed in present study may be related to the upstream MYB response elements. However this speculation has to be demonstrated by further cloning and function analyses of the promoter of *CmCADs*. Auxin (IAA) also plays a role in fruit development and ripening. Trainotti et al. [50] showed that there was an active crosstalk between IAA and ethylene that was important for the regulation of ripening. However, in case of melon, there were not reports about the inducing of ripening in mature fruit by IAA as done by ethylene. Of these, *CmCAD1* and *CmCAD5* expression were induced under IAA treatment (Figure 6; Figure S2), and other CAD genes were expressed at basal level but did not appear to be significantly regulated by IAA.

The promoter analysis of the five CAD genes revealed the presence ethylene responsive ERE motifs in *CmCAD3* and *CmCAD5* which have been shown to be responsive to ethylene treatment [51], [52] (Table S1). We found that the transcription level of *CmCADs* was obvious increase at 1 day after ethylene treatment and gradually decreased thereafter (Figure 7; Figure S3), apart from *CmCAD4*. However, *CmCADs* transcriptions were significantly suppressed by 1-MCP. Furthermore, ethylene was involved in lignification in *Brassica chinensis* and loquat flesh tissue by induced expression of *BcCAD1-1* and *BcCAD2* in loquat flesh and the expression of *EjCAD1* and *EjPOD* genes, respectively [47], [53]; while 1-MCP down-regulated them. Induced *GbCAD1* expression by ethylene may be related to enhancing PAL activity and subsequent product accumulation[42]. It is known to all that the biosynthesis of lignin in higher plants originates in the phenylalanine metabolic pathway. Therefore, the regulation of lignification and *CmCADs* expression of melon fruit tissue by ethylene during ripening may be related to the control in upstream of the phenylalanine metabolic pathway.

Additionally, we also identified several hormone-responsive cis-regulatory elements in the *CmCADs* promoter region, such as GARE, TATC-box, P-box (gibberellin), TCA-element (Salicylic acid), CCAAT-box, TGACG-motif and CGTCA-motif (MeJA)[19] (Table S1). A complex interplay of hormones is known to affect fruit development and ripening with auxin and GA being important during fruit expansion and ABA and ethylene for ripening [54]. In oriental sweet melon *(Cucumis melo* var. *makuwa* Makino), there are different ripening patterns within the fruit. The ripening of oriental sweet melon is initiated from the flesh and moves gradually towards the fruit cavity and the peel, and is earlier near the bottom and later at the carpopodium. The regulation pattern of ripening by hormones may selectively affect the expression of one or the other CADs. It also needs to be work out whether the regulation of hormones on different CAD gene members help in maintaining the net levels of CAD in fruit during ripening. These findings implied a complex hormonal regulation of the genes during fruit development and ripening and under stress conditions.

## Conclusions

Taken together, we identified five *CmCADs* in melon, phylogenetic analysis indicated that they belonged to four different groups, and *CmCAD* genes may function in process of fruit tissue lignification and in lignin biosynthesis in xylem and under different stress conditions through a CAD genes network. On the transcript level, differential *CmCADs* expression suggested tight adaptation of the fruit to the developmental events and biotic and abiotic stresses as well as cell division. Promoter sequence analysis and subcellular localization prediction implied that CAD genes had different functions. The five isoforms respond differently to ABA and IAA, in addition to ripening related hormone ethylene, suggesting distinct metabolic roles for these genes. Further studies related to biochemical characterization of these *CmCADs* in order to confirm their putative function is needed.

## Supporting Information

Figure S1 **Transcript abundance of CmCAD1, 2, 3, 4 and 5 in vegetative tissues and developing stages of melon fruit (1–48).** R, root; ML, mature leaf; DL, developing leaf; YS, young stem; PF, pistillate flower petal; SF, staminate flower petal. Stages and treatment were described in Section 2.5. RNA isolated from the samples. Expression analysis was carried out by semi-quantitative PCR using *18* *S* as an internal control.
(TIF)

Figure S2 **mRNA abundance of CmCAD1, 2, 3, 4, and 5 in mature green fruit after treatment with different hormonal.** Auxin and ABA (100 µM) treatments were given for 2 h as described in materials and methods section. Expression analysis was carried out by semi-quantitative PCR as described in Section 2.5 using actin as *18* *S* internal contrl.
(TIF)

Figure S3 **CmCAD1, 2, 3, 4 and 5 accumulation during different stages of ethylene and 1-MCP treatment induced ripening of melon by semi-quantitative PCR.** 1–12 indicate days after ethylene and 1-MCP treatment. The accumulation level of the genes in untreated melon fruit was control. *18* *S* was used as an internal control. All the treatments have been described in Section2.5.
(TIF)

Figure S4
**Amino acid sequence alignment of melon CmCAD1 (MELO3C019548P1^a^) and CmCAD2 (MELO3C018492P1^a^) with closely related sequences of other plants.** GenBank accession numbers are as follows: *Cucumis sativus* CsCAD11 (XP_004140716.1^b^), CsCAD12 (XP_004137094.1^b^), *Nicotiana tabacum* NtCAD1 (X62343^b^), *Populus tremuloides* PtCAD1 (AF217957^b^), *Arabidopsis thaliana* AtCAD4 (AY302081^b^), AtCAD5 (AY302082^b^), *Lolium perenne* LpCAD3 (AF010290^b^), *Festuca arundinacea* FaCAD1a (AF188292^b^), *Triticum aestivum* TaCAD1 (ADI59734.1^b^), *Oryza sativa* OsCAD2 (NP_001046132.1), *Zea mays* ZmCAD2 (ACG45271.1b), *Saccharum officinarum* SoCAD1 (AJ231135^b^), *Sorghum bicolor* SbCAD2 (AEM63607.1^b^), *Medicago sativa* MsCAD2 (AF083332^b^), *Eucalyptus globules* EgCAD2 (CAA46585.1^b^) and *Gossypium hirsutum* GhCAD1 (ABZ01817.1b). Conserved residues are shaded in black. The multi-domain architecture predicted by NCBI's CDD is marked: (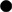) the black circle depicts the NADP binding site (aa 47–49, 52, 163, 167, 188–193, 211–212, 216, 232, 251–252, 254, 274–275, 298–300); (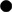)the grey circle depicts the substrate binding site (aa47, 49, 69, 95, 163, 300); (▽) white arrows depicts the catalytic Zn binding site (aa47, 69, 163); and (▾) black arrows depicts the structural Zn binding site (aa 100, 103, 106, 114). Dark grey shading indicates similar residues in seven out of eight of the sequences and clear grey shading indicates similar residues in five out of eight of the sequences. The letters following the accession numbers in the legend of the figure indicate the source database: (a) https://melonomics.net/ and (b) GenBank.(PPT)

Figure S5 **Amino acid sequence alignment of melon CmACD3 (MELO3C003735P2^a^) with closely related sequences of of other plants.** GenBank accession numbers are as follows: *Cucumis sativus* CsCAD6 (XP_004136373.1^b^), *Gossypium hirsutum* GhCAD3 (ACQ59091.1^b^), *Ricinus communis* RcCAD (XP_002510582.1^b^), *Theobroma cacao* TcCAD9 (EOY15101.1^b^), *Vitis vinifera* VvCAD6 (XP_002269356.1^b^), *Fragaria vesca* FvCAD6 (XP_004291336.1^b^), *Hordeum vulgare* HvCAD6 (BAK01962.1^b^) and *Sorghum bicolor* SbCAD6 (XP_002446076.1^b^). Conserved residues are shaded in black. The multi-domain architecture predicted by NCBI's CDD is marked: (•) the black circle depicts the NAD binding site (aa49–51, 54, 165, 169, 191–196, 214–215, 219, 235, 254–255, 257, 277–278, 301–303); (•)the grey circle depicts the substrate binding site (aa49, 51, 71, 97, 165, 303); (▽) white arrows depicts the catalytic Zn binding site (aa49, 71, 165); and (▾) black arrows depicts the structural Zn binding site (aa 102, 105, 108, 116). Dark grey shading indicates similar residues in seven out of eight of the sequences and clear grey shading indicates similar residues in five out of eight of the sequences. The letters following the accession numbers in the legend of the figure indicate the source database: (a) https://melonomics.net/ and (b) GenBank.
(PPT)

Figure S6
**Amino acid sequence alignment of melon CmCAD4 (MELO3C005809P1^a^) with closely related sequences of other plants.** GenBank accession numbers are as follows: *Cucumis sativus* CsCAD13 (XP004145884.1), CsCAD14 (XP004162965.1), *Sorghum bicolor* SbCAD4-2 (XP_002436635.1^b^), SbCAD4-3 (XP_002436634.1^b^), *Oryza sativa* OsCAD1 (AAN09864^b^), *Zea mays* ZmCAD4 (NP_001131273.1^b^), *Hordeum vulgare* HvCAD1A (BAJ84795.1^b^), HvCAD1B (BAJ98188.1^b^), *Triticum aestivum* TaCAD10 (TC172690^c^), *Gossypium hirsutum* GhCAD3 (ACQ59091.1^b^), *Ricinus communis* RcCAD (XP_002510582.1^b^), *Theobroma cacao* TcCAD9 (EOY15101.1^b^), *Vitis vinifera* VvCAD (CBI34634.3^b^), *Theobroma cacao* TcCAD4 (EOY23782.1^b^), *Glycine max* GmCAD1 (XP003543132.1^b^), *Cicer arietinum* CaCAD1 (XP004485621.1^b^) and *Arabidopsis thaliana* AtCAD1 (AY288079^b^). Conserved residues are shaded in black. The multi-domain architecture predicted by NCBI's CDD is marked: (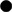) the black circle depicts the NAD binding site (aa49–51, 54, 165, 169, 191–196, 214–215, 219, 235, 254–255, 257, 277–278, 301–303); (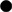)the grey circle depicts the substrate binding site (aa49, 51, 71, 97, 165, 303); (▽) white arrows depicts the catalytic Zn binding site (aa49, 71, 165); and (▾) black arrows depicts the structural Zn binding site (aa 102, 105, 108, 116). Dark grey shading indicates similar residues in seven out of eight of the sequences and clear grey shading indicates similar residues in five out of eight of the sequences. The letters following the accession numbers in the legend of the figure indicate the source database: (a) https://melonomics.net/, (b) GenBank and (c) http://compbio.dfci.harvard.edu/cgi-bin/tgi/Blast/index.cgi/1.(PPT)

Figure S7
**Amino acid sequence alignment of melon CmCAD5 (MELO3C023272P1^a^) with closely related sequences of other plants.** GenBank accession numbers are as follows: *Cucumis sativus* CsCAD9 (XP_004150677.1^b^), *Populus trichocarpa* PtCAD (XP_002300211.1^b^), *Populus trichocarpa* PtCAD6 (ABK94550.1^b^), *Populus tomentosa* PtCAD9 (AGU43751.1^b^), *Vitis vinifera* VvCAD9 (XP_002279832.1^b^), *Arabidopsis thaliana* AtCAD2 (AY302077^b^), AtCAD3 (AY302078^b^), AtCAD9 (AY302076^b^), *Medicago sativa* MsCAD1 (AF083333^b^) and PtSAD (AF273256^b^). Conserved residues are shaded in black. The multi-domain architecture predicted by NCBI's CDD is marked: (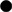) the black circle depicts the NAD binding site (aa50–52, 55, 166, 170, 191–196, 214–216, 219, 236, 254–255, 277–278, 301–303); (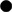)the grey circle depicts the substrate binding site (aa50, 52, 72, 98, 166, 303); (▽) white arrows depicts the catalytic Zn binding site (aa50, 72, 166); and (▾) black arrows depicts the structural Zn binding site (aa 103, 106, 109, 117). Dark grey shading indicates similar residues in seven out of eight of the sequences and clear grey shading indicates similar residues in five out of eight of the sequences. The letters following the accession numbers in the legend of the figure indicate the source database: (a) https://melonomics.net/and (b) GenBank.(PPT)

Table S1 **List and description of nucleotide motifs discovered in promoter region of melon CAD genes.**
(DOC)

Table S2 **CmCAD subcellular localization prediction.**
(DOC)
