# Supplementary figures and images for: The Cinnamyl Alcohol Dehydrogenase Gene Family in Melon (Cucumis melo L.): Bioinformatic Analysis and Expression Patterns
Source: PLoS One. 2014 Jul 14;9(7):e101730. doi: 10.1371/journal.pone.0101730 (PMC4096510; doi:10.1371/journal.pone.0101730)

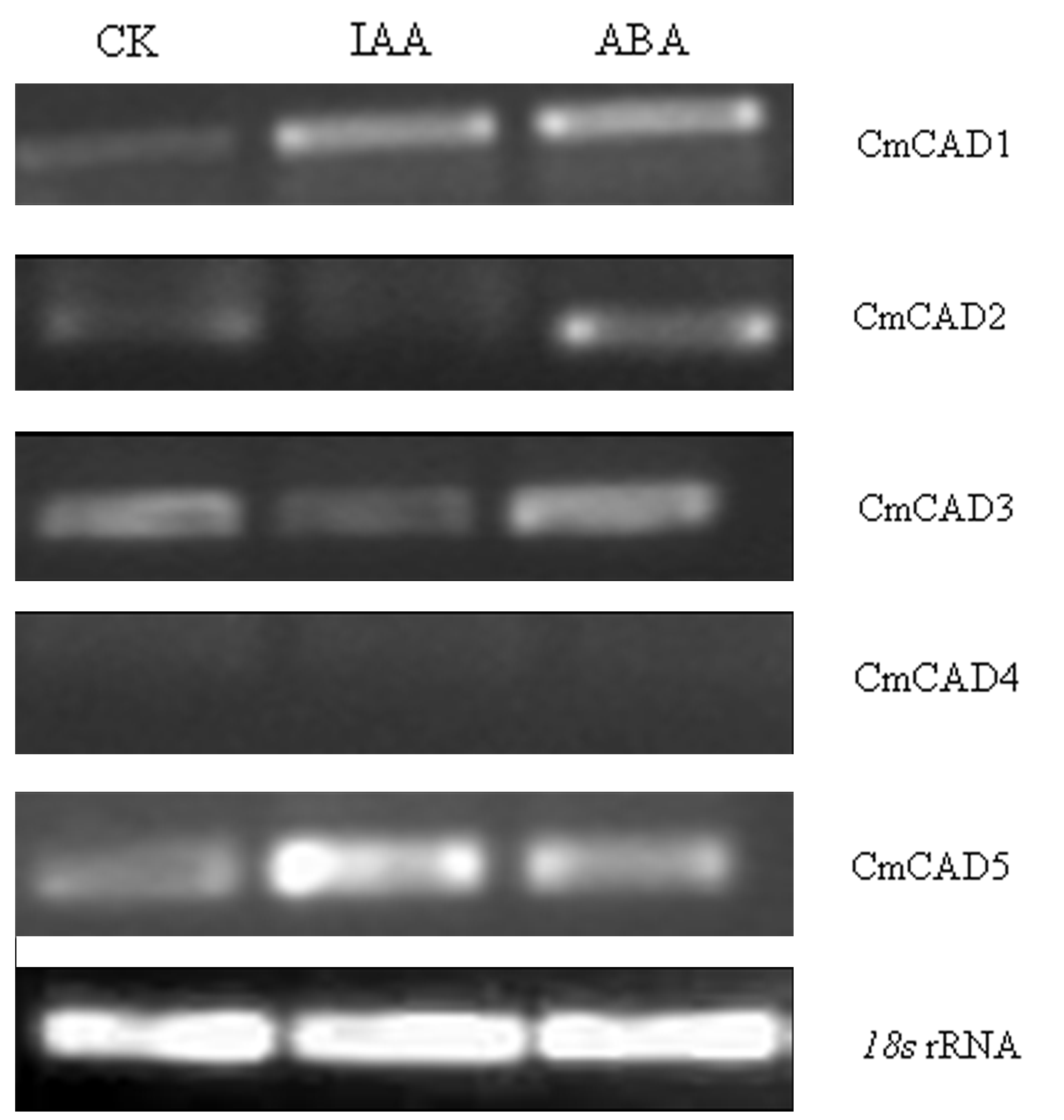

Supplement: Figure S2 — mRNA abundance of CmCAD1, 2, 3, 4, and 5 in mature green fruit after treatment with different hormonal. Auxin and ABA (100 µM) treatments were given for 2 h as described in materials and methods section. Expression analysis was carried out by semi-quantitative PCR as described in Section 2.5 using actin as 18 S internal contrl. (TIF) [file pone.0101730.s002.tif]

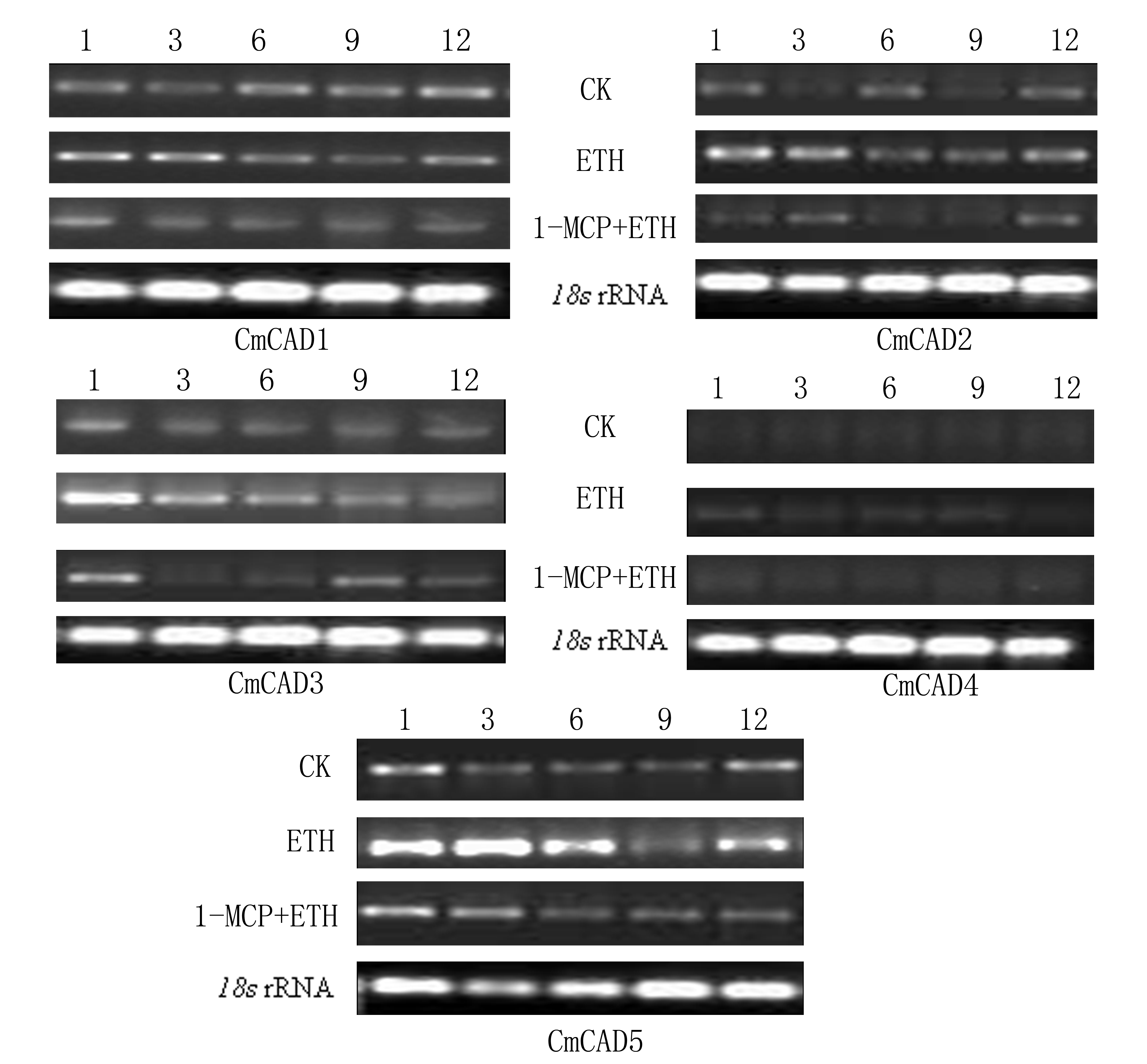

Supplement: Figure S3 — CmCAD1, 2, 3, 4 and 5 accumulation during different stages of ethylene and 1-MCP treatment induced ripening of melon by semi-quantitative PCR. 1–12 indicate days after ethylene and 1-MCP treatment. The accumulation level of the genes in untreated melon fruit was control. 18 S was used as an internal control. All the treatments have been described in Section2.5. (TIF) [file pone.0101730.s003.tif]
