# Supplementary material for: The Cinnamyl Alcohol Dehydrogenase Gene Family in Melon (Cucumis melo L.): Bioinformatic Analysis and Expression Patterns
Source: PLoS One. 2014 Jul 14;9(7):e101730. doi: 10.1371/journal.pone.0101730 (PMC4096510; doi:10.1371/journal.pone.0101730)
Supplement: Figure S4 — Amino acid sequence alignment of melon CmCAD1 (MELO3C019548P1a) and CmCAD2 (MELO3C018492P1a) with closely related sequences of other plants. GenBank accession numbers are as follows: Cucumis sativus CsCAD11 (XP_004140716.1b), CsCAD12 (XP_004137094.1b), Nicotiana tabacum NtCAD1 (X62343b), Populus tremuloides PtCAD1 (AF217957b), Arabidopsis thaliana AtCAD4 (AY302081b), AtCAD5 (AY302082b), Lolium perenne LpCAD3 (AF010290b), Festuca arundinacea FaCAD1a (AF188292b), Triticum aestivum TaCAD1 (ADI59734.1b), Oryza sativa OsCAD2 (NP_001046132.1), Zea mays ZmCAD2 (ACG45271.1b), Saccharum officinarum SoCAD1 (AJ231135b), Sorghum bicolor SbCAD2 (AEM63607.1b), Medicago sativa MsCAD2 (AF083332b), Eucalyptus globules EgCAD2 (CAA46585.1b) and Gossypium hirsutum GhCAD1 (ABZ01817.1b). Conserved residues are shaded in black. The multi-domain architecture predicted by NCBI's CDD is marked: () the black circle depicts the NADP binding site (aa 47–49, 52, 163, 167, 188–193, 211–212, 216, 232, 251–252, 254, 274–275, 298–300); ()the grey circle depicts the substrate binding site (aa47, 49, 69, 95, 163, 300); (▽) white arrows depicts the catalytic Zn binding site (aa47, 69, 163); and (▾) black arrows depicts the structural Zn binding site (aa 100, 103, 106, 114). Dark grey shading indicates similar residues in seven out of eight of the sequences and clear grey shading indicates similar residues in five out of eight of the sequences. The letters following the accession numbers in the legend of the figure indicate the source database: (a) https://melonomics.net/ and (b) GenBank. (PPT) [file pone.0101730.s004.ppt]

## Slide 1
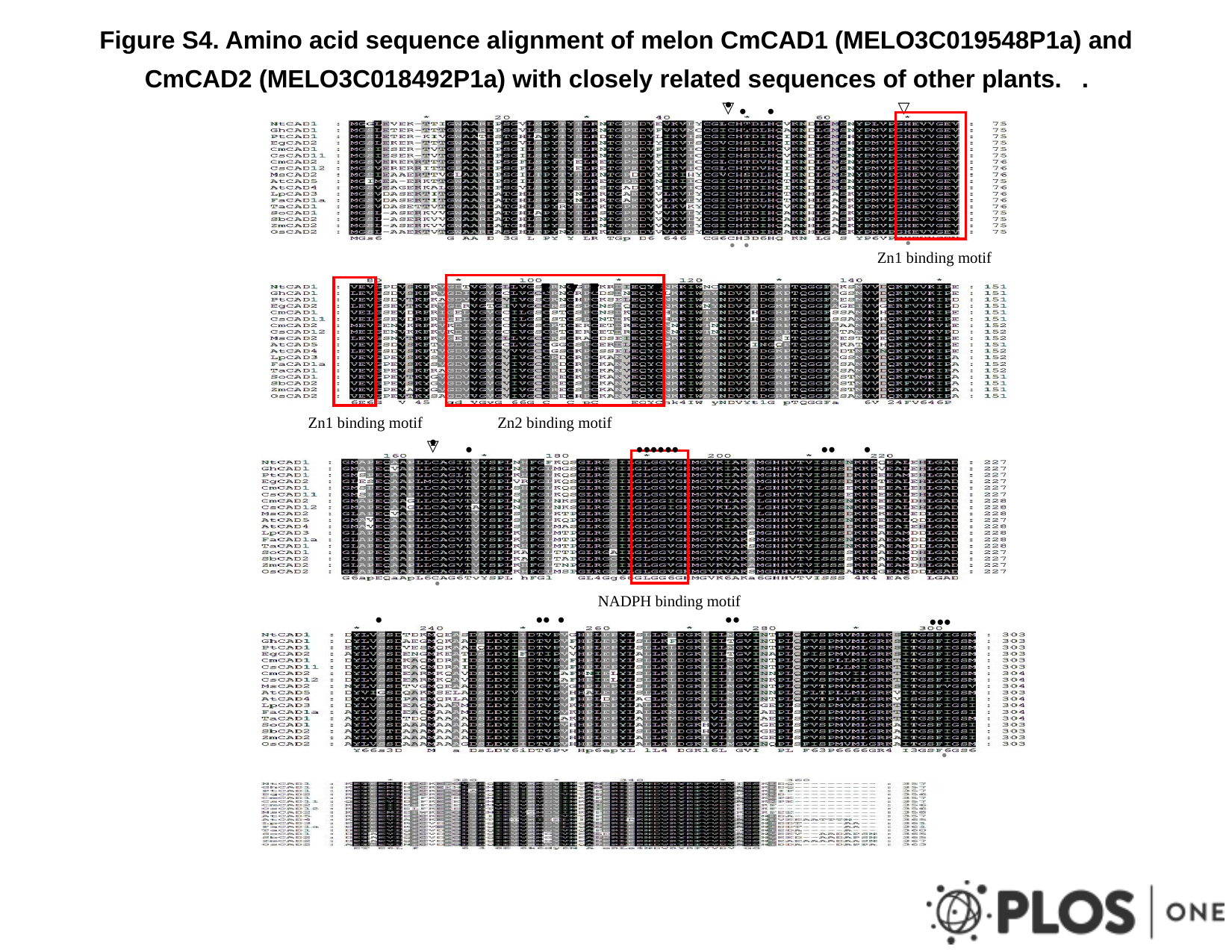

# Figure S4. Amino acid sequence alignment of melon CmCAD1 (MELO3C019548P1a) and CmCAD2 (MELO3C018492P1a) with closely related sequences of other plants. .
●
▽
▽
●
●
●
●
●
●
●
●
●
Zn1 binding motif
▼
▼
▼
▼
Zn1 binding motif
Zn2 binding motif
●
▽
●
●
●
●
●
●
●
●
●
●
●
●
●
NADPH binding motif
●
●
●
●
●
●
●
●
●
●
●
Figure.S4
