# Supplementary material for: The Cinnamyl Alcohol Dehydrogenase Gene Family in Melon (Cucumis melo L.): Bioinformatic Analysis and Expression Patterns
Source: PLoS One. 2014 Jul 14;9(7):e101730. doi: 10.1371/journal.pone.0101730 (PMC4096510; doi:10.1371/journal.pone.0101730)
Supplement: Figure S5 — Amino acid sequence alignment of melon CmACD3 (MELO3C003735P2a) with closely related sequences of of other plants. GenBank accession numbers are as follows: Cucumis sativus CsCAD6 (XP_004136373.1b), Gossypium hirsutum GhCAD3 (ACQ59091.1b), Ricinus communis RcCAD (XP_002510582.1b), Theobroma cacao TcCAD9 (EOY15101.1b), Vitis vinifera VvCAD6 (XP_002269356.1b), Fragaria vesca FvCAD6 (XP_004291336.1b), Hordeum vulgare HvCAD6 (BAK01962.1b) and Sorghum bicolor SbCAD6 (XP_002446076.1b). Conserved residues are shaded in black. The multi-domain architecture predicted by NCBI's CDD is marked: (•) the black circle depicts the NAD binding site (aa49–51, 54, 165, 169, 191–196, 214–215, 219, 235, 254–255, 257, 277–278, 301–303); (•)the grey circle depicts the substrate binding site (aa49, 51, 71, 97, 165, 303); (▽) white arrows depicts the catalytic Zn binding site (aa49, 71, 165); and (▾) black arrows depicts the structural Zn binding site (aa 102, 105, 108, 116). Dark grey shading indicates similar residues in seven out of eight of the sequences and clear grey shading indicates similar residues in five out of eight of the sequences. The letters following the accession numbers in the legend of the figure indicate the source database: (a) https://melonomics.net/ and (b) GenBank. (PPT) [file pone.0101730.s005.ppt]

## Slide 1
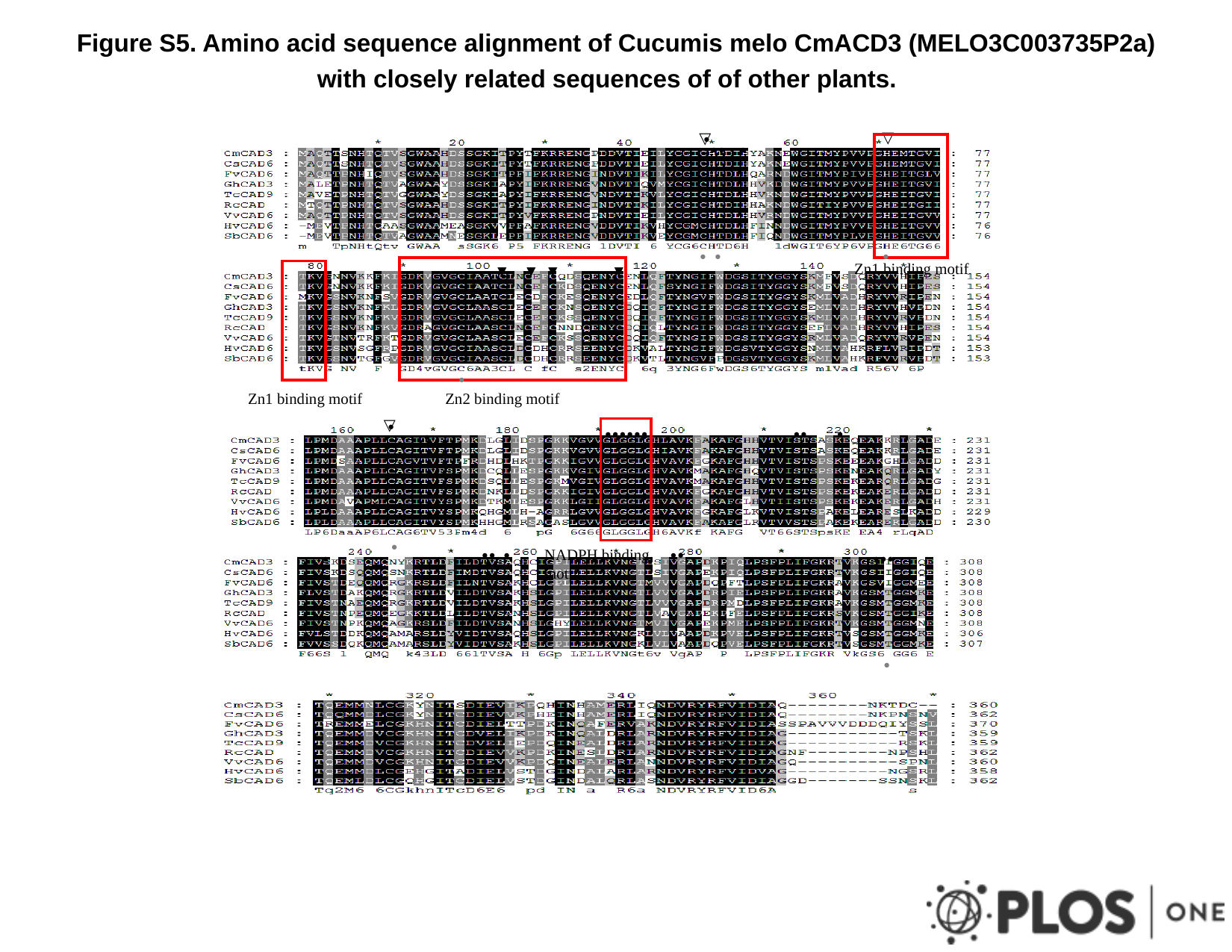

# Figure S5. Amino acid sequence alignment of Cucumis melo CmACD3 (MELO3C003735P2a) with closely related sequences of of other plants.
▽
▽
●
●
●
●
●
●
●
Zn1 binding motif
▼
▼
▼
▼
●
Zn2 binding motif
Zn1 binding motif
▽
●
●
●
●
●
●
●
●
●
●
●
●
NADPH binding motif
●
●
●
●
●
●
●
●
●
●
Figure.S5
