# Supplementary material for: The Cinnamyl Alcohol Dehydrogenase Gene Family in Melon (Cucumis melo L.): Bioinformatic Analysis and Expression Patterns
Source: PLoS One. 2014 Jul 14;9(7):e101730. doi: 10.1371/journal.pone.0101730 (PMC4096510; doi:10.1371/journal.pone.0101730)
Supplement: Figure S6 — Amino acid sequence alignment of melon CmCAD4 (MELO3C005809P1a) with closely related sequences of other plants. GenBank accession numbers are as follows: Cucumis sativus CsCAD13 (XP004145884.1), CsCAD14 (XP004162965.1), Sorghum bicolor SbCAD4-2 (XP_002436635.1b), SbCAD4-3 (XP_002436634.1b), Oryza sativa OsCAD1 (AAN09864b), Zea mays ZmCAD4 (NP_001131273.1b), Hordeum vulgare HvCAD1A (BAJ84795.1b), HvCAD1B (BAJ98188.1b), Triticum aestivum TaCAD10 (TC172690c), Gossypium hirsutum GhCAD3 (ACQ59091.1b), Ricinus communis RcCAD (XP_002510582.1b), Theobroma cacao TcCAD9 (EOY15101.1b), Vitis vinifera VvCAD (CBI34634.3b), Theobroma cacao TcCAD4 (EOY23782.1b), Glycine max GmCAD1 (XP003543132.1b), Cicer arietinum CaCAD1 (XP004485621.1b) and Arabidopsis thaliana AtCAD1 (AY288079b). Conserved residues are shaded in black. The multi-domain architecture predicted by NCBI's CDD is marked: () the black circle depicts the NAD binding site (aa49–51, 54, 165, 169, 191–196, 214–215, 219, 235, 254–255, 257, 277–278, 301–303); ()the grey circle depicts the substrate binding site (aa49, 51, 71, 97, 165, 303); (▽) white arrows depicts the catalytic Zn binding site (aa49, 71, 165); and (▾) black arrows depicts the structural Zn binding site (aa 102, 105, 108, 116). Dark grey shading indicates similar residues in seven out of eight of the sequences and clear grey shading indicates similar residues in five out of eight of the sequences. The letters following the accession numbers in the legend of the figure indicate the source database: (a) https://melonomics.net/, (b) GenBank and (c) http://compbio.dfci.harvard.edu/cgi-bin/tgi/Blast/index.cgi/1. (PPT) [file pone.0101730.s006.ppt]

## Slide 1
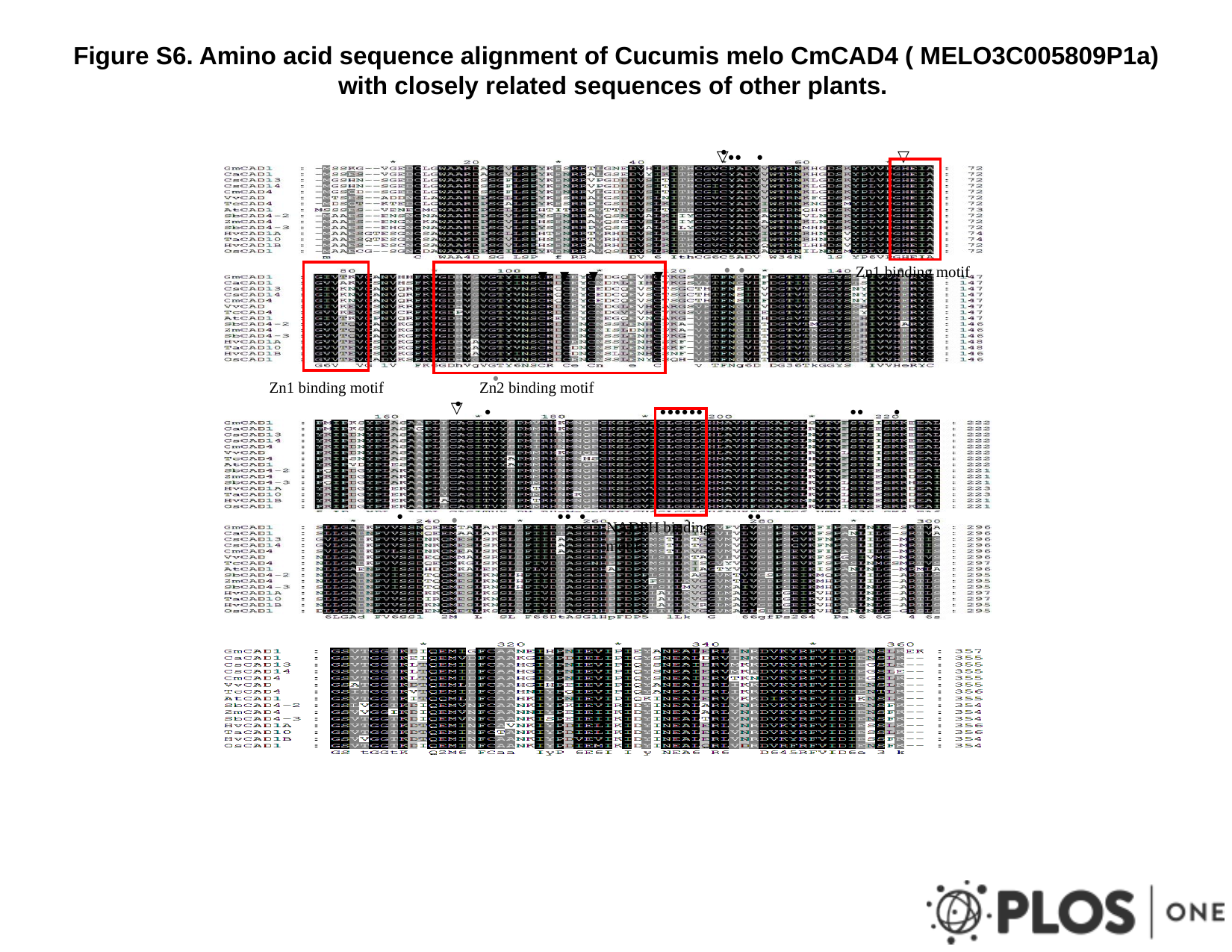

# Figure S6. Amino acid sequence alignment of Cucumis melo CmCAD4 ( MELO3C005809P1a) with closely related sequences of other plants.
●
▽
▽
●
●
●
Zn1 binding motif
●
●
●
▼
▼
▼
▼
●
Zn1 binding motif
Zn2 binding motif
●
▽
●
●
●
●
●
●
●
●
●
●
●
●
●
●
●
●
●
NADPH binding motif
