# Supplementary material for: The Cinnamyl Alcohol Dehydrogenase Gene Family in Melon (Cucumis melo L.): Bioinformatic Analysis and Expression Patterns
Source: PLoS One. 2014 Jul 14;9(7):e101730. doi: 10.1371/journal.pone.0101730 (PMC4096510; doi:10.1371/journal.pone.0101730)
Supplement: Figure S7 — Amino acid sequence alignment of melon CmCAD5 (MELO3C023272P1a) with closely related sequences of other plants. GenBank accession numbers are as follows: Cucumis sativus CsCAD9 (XP_004150677.1b), Populus trichocarpa PtCAD (XP_002300211.1b), Populus trichocarpa PtCAD6 (ABK94550.1b), Populus tomentosa PtCAD9 (AGU43751.1b), Vitis vinifera VvCAD9 (XP_002279832.1b), Arabidopsis thaliana AtCAD2 (AY302077b), AtCAD3 (AY302078b), AtCAD9 (AY302076b), Medicago sativa MsCAD1 (AF083333b) and PtSAD (AF273256b). Conserved residues are shaded in black. The multi-domain architecture predicted by NCBI's CDD is marked: () the black circle depicts the NAD binding site (aa50–52, 55, 166, 170, 191–196, 214–216, 219, 236, 254–255, 277–278, 301–303); ()the grey circle depicts the substrate binding site (aa50, 52, 72, 98, 166, 303); (▽) white arrows depicts the catalytic Zn binding site (aa50, 72, 166); and (▾) black arrows depicts the structural Zn binding site (aa 103, 106, 109, 117). Dark grey shading indicates similar residues in seven out of eight of the sequences and clear grey shading indicates similar residues in five out of eight of the sequences. The letters following the accession numbers in the legend of the figure indicate the source database: (a) https://melonomics.net/and (b) GenBank. (PPT) [file pone.0101730.s007.ppt]

## Slide 1
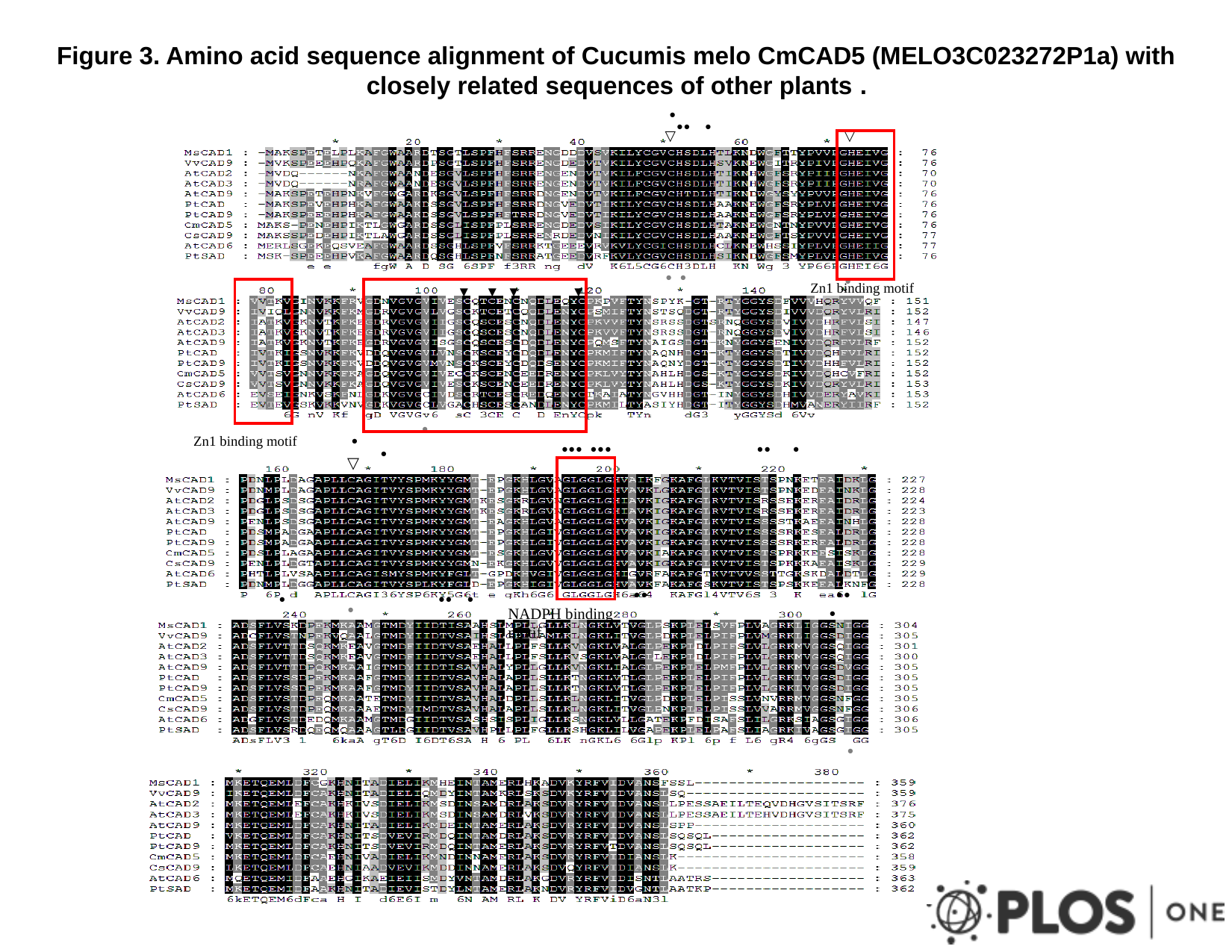

# Figure 3. Amino acid sequence alignment of Cucumis melo CmCAD5 (MELO3C023272P1a) with closely related sequences of other plants .
●
●
●
●
▽
▽
●
●
●
Zn1 binding motif
▼
▼
▼
▼
●
Zn1 binding motif
●
●
●
●
●
●
●
●
●
●
●
▽
●
●
●
●
●
●
●
●
●
NADPH binding motif
●
●
Figure.S7
