# Supplementary material for: The Cinnamyl Alcohol Dehydrogenase Gene Family in Melon (Cucumis melo L.): Bioinformatic Analysis and Expression Patterns
Source: PLoS One. 2014 Jul 14;9(7):e101730. doi: 10.1371/journal.pone.0101730 (PMC4096510; doi:10.1371/journal.pone.0101730)
Supplement: Table S1 — List and description of nucleotide motifs discovered in promoter region of melon CAD genes. (DOC) [file pone.0101730.s008.doc]

Table S1: List and description of nucleotide motifs discovered in promoter region of melon CAD genes

| Motif name | Gene name | Motif sequence | Motif database | Motif function |
| --- | --- | --- | --- | --- |
| TC-rich repeats | *CmCAD1* | ATTTTCTTCA;  GTTTTCTTAC | PlantCARE | Cis-acting element involved in defense and stress responsiveness |
|  | *CmCAD2* |  |  |  |
|  | *CmCAD4* |  |  |  |
|  | *CmCAD5* |  |  |  |
| BOX-W1(W BOX) | *CmCAD3* | TTGACC | PlantCARE | Fungal elicitor responsive element |
|  | *CmCAD5* |  |  |  |
| ARE | *CmCAD1* | TGGTTT | PlantCARE | Cis-acting element involved in hypoxia stress responsiveness |
|  | *CmCAD2* |  |  |  |
|  | *CmCAD3* |  |  |  |
|  | *CmCAD4* |  |  |  |
| CGTCA-motif | *CmCAD5* | CGTCA, TGACG | Plant CARE | Cis-acting regulatory element involved in the MeJA-responsiveness |
| TGACG-motif | *CmCAD3* | TGACG | Plant CARE | Cis-acting regulatory element involved in the MeJA-responsiveness |
| ELI-box3 | *CmCAD1* | AAACCAATT | PlantCARE | Elicitor responsive element |
| TA-rich region | *CmCAD2* |  | PlantCARE | enhancer |
| CCAAT-box | *CmCAD3* | CAACGG | Plant CARE | Cis-acting regulatory element involved in the MeJA-responsiveness |
| HSE | *CmCAD2* | AAAAAATTTC | PlantCARE | Cis-acting element involved in heat stress responsiveness |
|  | *CmCAD3* | AAAAAATTTC |  |  |
|  | *CmCAD4* | AAAATTTAAT |  |  |
|  | *CmCAD5* | AAAAAATGTC |  |  |
| MBS | *CmCAD4* | CAACTG,TAACTG | PlantCARE | MYB binding site involved in drought-inducibility |
| GARE-motif | *CmCAD2* | TCTGTTG | PlantCARE | Cis-acting element involved in the gibberellin responsiveness |
|  | *CmCAD3* |  |  |  |
|  | *CmCAD4* |  |  |  |
|  | *CmCAD5* |  |  |  |
| TATC-box | *CmCAD4* | TATCCCA | PlantCARE | Cis-acting element involved in the gibberellin responsiveness |
| P-box | *CmCAD2* | CCTTTTG | PlantCARE | Cis-acting element involved in the gibberellin responsiveness |
|  | *CmCAD4* |  |  |  |
| ABRE | *CmCAD1* | ACGTGGC | PlantCARE | Cis-acting element involved in the abscisic acid responsiveness |
|  | *CmCAD2* | ACGTGGC |  |  |
|  | *CmCAD3* | CGCACGTGTC |  |  |
|  | *CmCAD5* | CACGTG |  |  |
| WUN-motif | *CmCAD3* | TCATTACAA | PlantCARE | Wound-responsive element |
| ERE | *CmCAD3* | ATTTCAAA | PlantCARE | Ethylene-responsive |
|  | *CmCAD5* |  |  |  |
| TGA | *CmCAD1* | AACGAC | PlantCARE | Auxin responsive |
|  | *CmCAD5* |  |  |  |
| TCA-element | *CmCAD1* | CCATCTTTTT | PlantCARE | Salicylic acid response |
|  | *CmCAD2* | TCAGAAGAGA |  |  |
|  | *CmCAD5* | TCAGAAAAGG |  |  |
