# Supplementary material for: The Cinnamyl Alcohol Dehydrogenase Gene Family in Melon (Cucumis melo L.): Bioinformatic Analysis and Expression Patterns
Source: PLoS One. 2014 Jul 14;9(7):e101730. doi: 10.1371/journal.pone.0101730 (PMC4096510; doi:10.1371/journal.pone.0101730)
Supplement: Table S2 — CmCAD subcellular localization prediction. (DOC) [file pone.0101730.s009.doc]

Table S2 CmCAD subcellular localization prediction

| CAD | LocTree3 | | CELLOV 2.5 | | Pslpre | | | Wolfprort | |
| --- | --- | --- | --- | --- | --- | --- | --- | --- | --- |
|  | Score | Localization Class | Score | Localization Class | RI | Expected Accuracy | Localization Class | Score | Localization Class |
| CmCAD1 | 35 | cytoplasm | 3.344 | cytoplasm | 4 | 90.2% | cytoplasm | 11 | cytoplasm |
| CmCAD2 | 36 | cytoplasm | 4.196 | cytoplasm | 5 | 98.1% | cytoplasm | 8 | cytoplasm |
| CmCAD3 | 40 | cytoplasm | 2.402 | cytoplasm | 3 | 71.1% | cytoplasm | 7 | Peroxisomal |
| CmCAD4 | 37 | cytoplasm | 1.696 | cytoplasm | 2 | 68.1% | cytoplasm | 7 | cytoplasm |
| CmCAD5 | 50 | cytoplasm | 3.223 | cytoplasm | 3 | 71.1% | cytoplasm | 9 | cytoplasm |
